# Khdrbs1 drives re-differentiation of bipotential progenitor cells by inhibiting p53 in zebrafish biliary-mediated liver regeneration

**DOI:** 10.1242/dev.204266

**Published:** 2025-02-28

**Authors:** Kai Gang, Qi Chen, Junhui Sun, Tingwei Zhang, Pengcheng Cai, Rui Ni, Jianlong Ma

**Affiliations:** ^1^ Institute of Developmental Biology and Regenerative Medicine, Southwest University, Chongqing 400715, China; ^2^ State Key laboratory of Genetic Engineering, School of Life Sciences, Liver Cancer Institute of Zhongshan Hospital, Fudan University, Shanghai 200438, China

**Keywords:** Liver injury, Biliary epithelial cell, Transdifferentiation, Cell proliferation, *Sam68*

## Abstract

After severe liver injury, biliary epithelial cells (BECs) undergo de-differentiation into bipotential progenitor cells (BPPCs), which subsequently re-differentiate into nascent hepatocytes and BECs to accomplish liver regeneration. However, the crucial factors governing the re-differentiation process of BPPCs remain largely unknown. Here, using a zebrafish model of severe liver injury, we observed specific expression of *khdrbs1a* and *khdrbs1b* (collectively known as *khdrbs1*) in BPPCs through single-cell RNA analyses and fluorescence *in situ* hybridization. Subsequently, to eliminate the genetic compensation, we generated a CRISPR/dead Cas9-mediated system for interfering with *khdrbs1* in BECs, which caused defective liver regeneration and impaired re-differentiation of BPPCs. Furthermore, the *khdrbs1*^−/−^ mutant displayed impaired proliferation and re-differentiation of BPPCs during liver regeneration. Mechanistically, p53 signaling was activated in response to the loss of *khdrbs1*, and *tp53* mutation partially rescued the defective liver regeneration of the *khdrbs1*^−/−^ mutant. In summary, we conclude that Khdrbs1 promotes the re-differentiation of BPPCs in part by inhibiting p53 activation during biliary-mediated liver regeneration in zebrafish.

## Teaser

**Summary:** Loss of Khdrbs1 activates p53 and impairs liver regeneration after severe liver injury in zebrafish.

## INTRODUCTION

The liver has a fantastic regenerative capacity to support the recovery of its functions (Ben-Moshe et al., 2022). Liver diseases, such as cirrhosis and acute liver failure, typically result from disrupted homeostasis and are often associated with insufficient functional hepatocytes or impaired recovery (Michalopoulos and Bhushan, 2021). Hepatocytes and biliary epithelial cells (BECs) are the two major functional cell populations associated with maintaining liver homeostasis. Various hepatocyte subtypes have been reported to contribute to the maintenance of liver homeostasis and rapid compensation for liver injury (Font-Burgada et al., 2015; He et al., 2021; Lin et al., 2018; Wei et al., 2021). However, most liver diseases frequently accompany ductular reaction, whereby BECs proliferate and bile ducts are increased, with some cells expressing features of both BECs and hepatocytes (Raven and Forbes, 2018). It remains an interesting question whether ductular reaction is conducive to or has the potential for improving liver repair and regeneration. Previous studies demonstrate that BECs can transdifferentiate into hepatocytes via the ductular reaction after severe liver damage in mice (Deng et al., 2018; Pu et al., 2023).

Thus, the promotion of transdifferentiation from BEC into hepatocyte represents a promising clinical therapy for individuals with end-stage liver diseases. To validate the role of BECs in liver regeneration and eliminate the contribution from hepatocytes, the nitroreductase (NTR)/metronidazole (Mtz) system has been developed to ablate hepatocytes and induce severe liver injury in zebrafish (*Danio rerio*), with Mtz acting as a prodrug that is converted into a cytotoxic substance by NTR (Curado et al., 2007). In this model, the liver requires at least 72 h to regenerate to its pre-injury level (Cai et al., 2021; Choi et al., 2014). Following extensive hepatocyte ablation, BECs proliferate and facilitate recovery from severe liver injury by de-differentiating into bipotential progenitor cells (BPPCs), which subsequently re-differentiate into nascent hepatocytes (Choi et al., 2014; He et al., 2014), a process known as biliary-mediated liver regeneration. BPPCs express hepatoblast markers, such as *sox9b* (*SRY-box transcription factor 9b*), *foxa3* (*forkhead box A3*), and *hhex* (*hematopoietically expressed homeobox*), and exhibit mTORC1 activity (He et al., 2019) after hepatocyte ablation. However, the essential factors governing the re-differentiation process of BPPCs into nascent hepatocytes remain largely unknown.

Our previous single-cell RNA-sequencing data (Cai et al., 2021) identified a clear expression pattern of *khdrbs1a* and *khdrbs1b* (collectively called *khdrbs1*), orthologous to human *KHDRBS1* (KH domain-containing, RNA-binding, signal transduction-associated protein 1; also known as SAM68), in BPPCs during zebrafish biliary-mediated liver regeneration. In mammals, KHDRBS1 functions in both RNA binding and signal transduction, by shuttling between the nucleus and cytoplasm, and influences a range of cellular processes, including neuronal homeostasis (Iijima et al., 2011), adipogenesis (Song and Richard, 2015), spermatogenesis (Paronetto et al., 2011), and even tumorigenesis (Benoit et al., 2017; Fu et al., 2016). KHDRBS1 also functions as an adaptor of inflammatory signaling (Gowd et al., 2024) and even plays roles in transcription as a co-activator with AR (Rajan et al., 2008) and p53 (TP53) (Li and Richard, 2016), as well as a repressor of CBP (CREBBP) (Benoit et al., 2017). Clinically, KHDRBS1 serves as a potential prognostic biomarker for hepatocellular carcinoma (Fan et al., 2024; Zhang et al., 2015). Moreover, KHDRBS1 deficiency can improve the recovery of the injured artery by alleviating the NF-κB signaling-mediated pro-inflammatory response (Han et al., 2019), and expression of KHDRBS1 in diabetic hyperglycemia promotes hepatic gluconeogenesis by reducing CRTC2 ubiquitination (Qiao et al., 2021). Nevertheless, the role of KHDRBS1 in liver regeneration remains poorly understood.

In this study, specific inactivation of *khdrbs1* in BECs or *khdrbs1* genetic mutation led to defective phenotypes in biliary-mediated liver regeneration and activated p53 signaling, impairing the proliferation and re-differentiation of BPPCs. Thus, we demonstrate that Khdrbs1 plays an essential role in promoting biliary-mediated liver regeneration by regulating p53 signaling.

## RESULTS AND DISCUSSION

### The expression of *khdrbs1* is upregulated in BPPCs during biliary-mediated liver regeneration

**Fig. 1.**
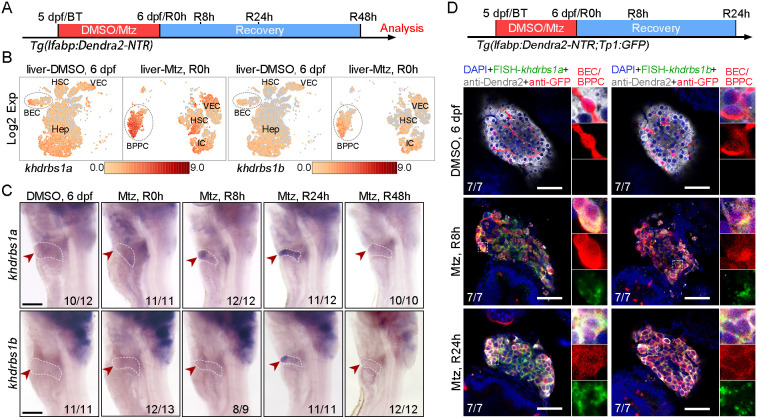
***khdrbs1a* and *khdrbs1b* are expressed in BPPCs during liver regeneration.** (A) Scheme showing the DMSO or Mtz treatment and analysis stages during liver regeneration. (B) UMAP showing *khdrbs1a* or *khdrbs1b* expression (overlaid in red scale) in various liver cell types at 6 dpf and R0 h. BEC, biliary epithelial cell; BPPC, bipotential progenitor cell; Hep, hepatocyte; HSC, hepatic stellate cell; IC, immune cell; VEC, vascular endothelial cell. Black dashed circle indicates BEC/BPPC. (C) WISH images showing the expression patterns of *khdrbs1a* or *khdrbs1b* during liver regeneration at 6 dpf, R0 h, R8 h, R24 h, and R48 h. Red arrowheads and white dashed outlines indicate livers. Scale bars: 100 μm. (D) Confocal images showing the expression of *khdrbs1a* or *khdrbs1b* in BECs or BPPCs at 6 dpf, R8 h, and R24 h by FISH in *Tg(lfabp:Dendra2-NTR; Tp1:GFP)* in which hepatocytes and BECs are labeled, respectively. Insets show magnified views of regions of interest. Schematic at the top shows the Mtz treatment and analysis stages. Numbers at the bottom of image panels indicate the proportion of larvae exhibiting the expression shown. Scale bars: 50 μm.

From 5 days post-fertilization (dpf), zebrafish liver is similar to that of an adult (Chu and Sadler, 2009). Using a zebrafish severe liver injury model, the *Tg(lfabp:Dendra2-NTR)* line (He et al., 2014), liver injury was induced from 5 dpf (designated ‘BT’, before Mtz treatment) to 6 dpf, followed by regeneration of nascent hepatocytes derived from BECs (Fig. S1A-C). Based on previously reported single-cell RNA-sequencing data from wild-type livers at 6 dpf and time point ‘regeneration 0 h’ (R0 h) (Cai et al., 2021; accessible at National Center for Biotechnology Information Sequence Read Archive, under BioProject PRJNA975724), the expression of BEC/BPPC markers, proliferation markers, and hepatocyte markers were used to characterize cells (Fig. S2A-C). We further observed that both *khdrbs1* paralogs were enriched in BPPCs at R0 h after hepatocyte ablation, particularly *khdrbs1a*, in this previously published dataset (Fig. 1A,B). To investigate the expression pattern of *khdrbs1* in liver regeneration further, we performed whole-mount *in situ* hybridization (WISH) from R0 h to R48 h. WISH results showed that *khdrbs1a* expression was apparently upregulated in the liver at R8 h and R24 h during liver regeneration, while the expression of *khdrbs1b* showed slightly elevated at R8 h and notably increased at R24 h (Fig. 1C). Quantitative real-time polymerase chain reaction (qPCR) analysis of *khdrbs1a* expression indicated a significant upregulation at both R0 h and R8 h, whereas *khdrbs1b* expression exhibited a marked increase at R8 h (Fig. S3A,B). Fluorescence *in situ* hybridization (FISH) analysis of *khdrbs1* also showed mainly increased expression in Anxa4 (Annexin A4)-labeled BECs/BPPCs in response to severe liver injury at Mtz-16 h and R0 h (Fig. S3C,D, Fig. S4A-C). We further analyzed the expression pattern of *khdrbs1* in BPPCs at R8 h and R24 h using the *Tg(Tp1:GFP)* line to label intrahepatic BECs during liver regeneration. FISH antibody staining assays revealed that both *khdrbs1a* and *khdrbs1b* were mainly expressed in BPPCs co-expressed GFP and Dendra2 at R8 h and R24 h (Fig. 1D; Fig. S4D). Additionally, the expression of *khdrbs1* in liver region was initiated at 56 hours post-fertilization (hpf) and ceased by 4 dpf during liver development (Fig. S5A). These data imply that elevated *khdrbs1* may participate in biliary-mediated liver regeneration.

### Specific inactivation of Khdrbs1 in BECs results in impaired biliary-mediated liver regeneration

CRISPR/Cas9 technology has been widely applied in genome editing. We used this method to knock out *khdrbs1a* and *khdrbs1b* in zebrafish. A *khdrbs1a* mutant was generated by knockout of two loci, causing the insertion of 1 base pair (bp) in exon 1 and an indel (Δ8, +6 bp) in exon 6 (Fig. S5B). A *khdrbs1b* mutant was generated by editing exon 3, resulting in an indel (Δ3, +87 bp) (Fig. S5C). These frameshift mutations caused premature termination codons in open reading frames. In addition, *khdrbs1a* expression was downregulated in *khdrbs1a*^−/−^ mutants at 3.5 dpf, as was *khdrbs1b* expression in *khdrbs1b*^−/−^ mutants (Fig. S5D,E), suggesting nonsense-mediated mRNA decay.

**Fig. 2.**
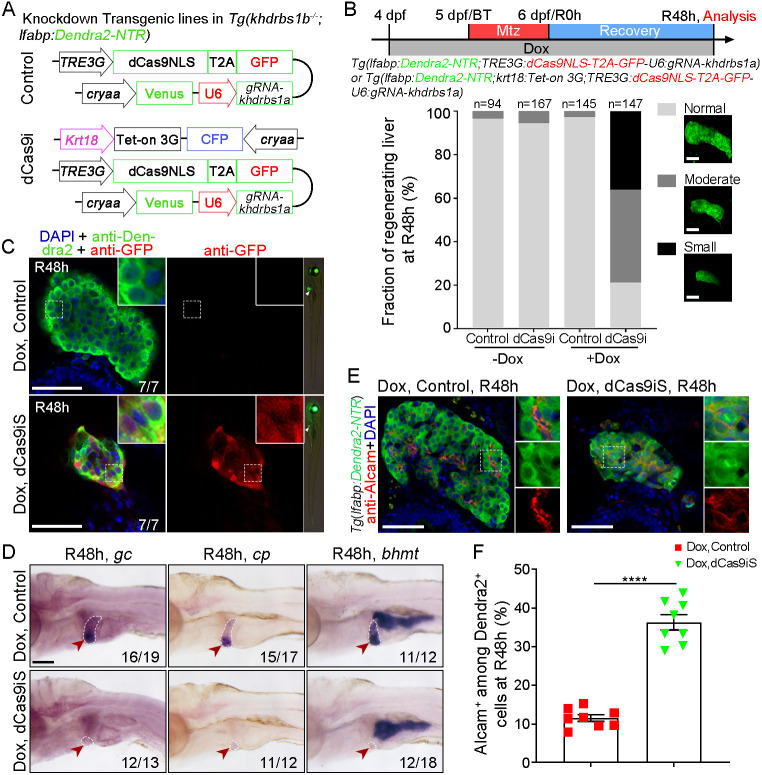
**Specific inactivation of *khdrbs1* in BECs causes impaired biliary-mediated liver regeneration.** (A) Schematic showing CRISPR/dead Cas9-mediated interference (dCas9i) to knock down *khdrbs1* in BECs, combined with doxycycline (Dox)-induced Tet-on3G/TRE3G system and constructed in the background of *khdrbs1b*^−/−^ mutants. (B) Scheme showing the treatment of Dox and Mtz and then analysis at R48 h. Graph shows the percentage of regenerating livers of the control and dCas9i groups at R48 h with or without Dox. Images show examples of the normal, moderate and small categories. ‘Normal’ represents a regenerating liver size comparable to that of wild type. ‘Small’ represents a severe deficiency of liver regeneration compared to wild type. (C) Right-hand images: Epifluorescence images showing the body shape and regenerating livers (white arrowheads). Main panels: Confocal images showing the regenerating livers of control and small regenerated liver in the dCas9i group (dCas9iS) at R48 h. Insets show magnified views of regions of interest. (D) WISH images showing the expressions of *gc*, *cp*, and *bhmt* at R48 h. Red arrowheads and white dashed outlines indicate livers. Scale bar: 100 μm. (E) Confocal images showing the expression of Alcam and Dendra2 at R48 h. Scale bars: 50 μm. (F) Quantification of Alcam^+^ among Dendra2^+^ cells in regenerating livers at R48 h. Error bars represent s.e.m. ****P<0.0001 (two-tailed, unpaired *t*-test; control group, n=7; dCas9iS subgroup, *n*=8.). Numbers at the bottom of images indicate the proportion of larvae exhibiting the expression shown.

However, *khdrbs1a*^−/−^ or *khdrbs1b*^−/−^ mutants showed normal liver regeneration after hepatocyte ablation (Fig. S6A,B), suggesting potential genetic compensation or functional redundancy between the two paralogs. We attempted to disrupt both paralogs of *khdrbs1* during liver regeneration using CRISPR/dead Cas9 technology, which has been utilized to promote or repress gene transcription (Gilbert et al., 2013). We constructed a BEC-specific CRISPR/dead Cas9-mediated interference system (dCas9i) driven by Tet-on 3G/TRE3G and the BEC promoter *krt18* to knock down *khdrbs1a* expression in *khdrbs1b*^−/−^ mutants from 4 dpf (Fig. 2A). Indeed, a decrease in *khdrbs1a* expression was induced by doxycycline (Dox) at R24 h in regenerating livers of the dCas9i group (Fig. S6C), indicating that dCas9i is an efficient system for repressing *khdrbs1a* expression during biliary-mediated liver regeneration. After Dox treatment and hepatocyte ablation, liver regeneration was compromised in the dCas9i group at R48 h compared with the control group (Fig. 2B). The data indicated that approximately 36.0% of larvae exhibited a small regenerated liver (abbreviated as dCas9iS subgroup), and about 48.6% of larvae displayed moderate liver regeneration in the dCas9i group compared to control group (Fig. 2B). Furthermore, the regenerating livers of the dCas9iS subgroup co-expressed Dendra2 and GFP induced by Dox at R48 h, demonstrating that this dCas9i system is BEC specific for biliary-mediated liver regeneration (Fig. 2C). The mature hepatocyte markers *gc* (*GC vitamin D binding protein*), *cp* (*ceruloplasmin*), and *bhmt* (*betaine-homocysteine methyltransferase*) were downregulated in the dCas9iS group at R48 h, indicating defective liver regeneration after knockdown of *khdrbs1a* (Fig. 2D). An increased proportion of the cells labeled with the biliary marker Alcam (activated leukocyte cell adhesion molecule) was observed among Dendra2-expressing cells in the dCas9iS subgroup at R48 h, suggesting impaired re-differentiation of BPPCs after loss of Khdrbs1(Fig. 2E,F). Therefore, the outcomes of our dCas9i system are reliable and authentic, although the small regenerated liver is not up to 50.0% in the dCas9i group. We attribute this to the expression level or activity of induced dead Cas9, implying that improvements could be made to this method.

### Genetic mutation of *khdrbs1* impairs biliary-mediated liver regeneration

**Fig. 3.**
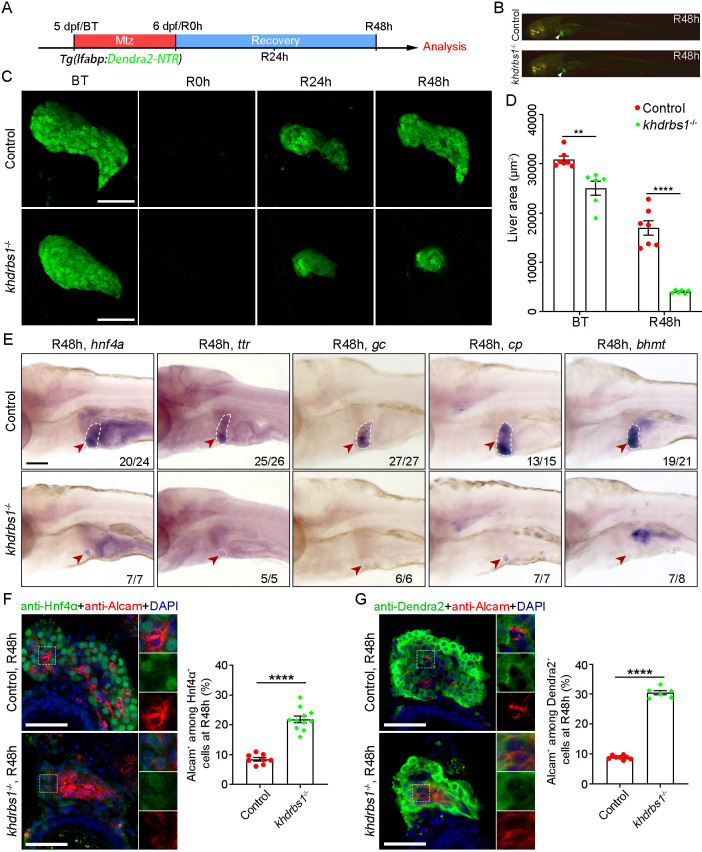
**Loss of *khdrbs1* impairs the re-differentiation of BPPCs during biliary-mediated liver regeneration.** (A) Scheme showing the stage of Mtz treatment and analysis at R48 h. (B) Epifluorescence images showing the body shape and regenerating livers (white arrowheads) of the control and *khdrbs1*^−/−^ mutant at R48 h. (C) Confocal projection images showing livers of the control and *khdrbs1*^−/−^ mutant at BT (before treatment), R0 h, R24 h, and R48 h. Scale bars: 100 μm. (D) Quantification of liver area in the control and *khdrbs1*^−/−^ mutant at BT and R48 h. 5 dpf: control, *n*=7; *khdrbs1*^−/−^, *n*=6; R48 h: control, *n*=7; *khdrbs1*^−/−^, *n*=6. (E) WISH images showing expression of the hepatocyte markers *hnf4α*, *ttr*, *gc*, *cp*, and *bhmt* in the control and *khdrbs1*^−/−^ mutant at R48 h. Red arrowheads and white dashed outlines indicate livers. Numbers indicate the proportion of larvae exhibiting the expression/phenotype shown. Scale bar: 100 μm. (F,G) Confocal images and quantification showing the expression of Alcam and Hnf4α or Alcam and Dendra2 at R48 h. Insets show magnified views of regions of interest. F: control, *n*=8; *khdrbs1*^−/−^, *n*=11; G: control, *n*=8; *khdrbs1*^−/−^, *n*=6. Error bars represent s.e.m. **P<0.01, ****P<0.0001 (two-tailed, unpaired *t*-test). Scale bars: 50 μm.

To avoid genetic redundancy and validate the role of Khdrbs1 at the genetic level, a double mutant (abbreviated as *khdrbs1*^−/−^ mutant) was generated from parents that were *khdrbs1b*^−/−^ mutants carrying a heterozygous mutation of *khdrbs1a* (Fig. S5F). After liver injury, the *khdrbs1*^−/−^ mutant exhibited defective liver regeneration (Fig. 3A,B). The *khdrbs1*^−/−^ mutant liver was slightly smaller than the control liver at 5 dpf. However, the regenerating liver of the *khdrbs1*^−/−^ mutant was dramatically smaller than that of the control at R48 h and liver area was about one-third of the control (Fig. 3C,D). Furthermore, the hepatocyte markers *hnf4a* (*hepatocyte nuclear factor 4, alpha*), *ttr* (*transthyretin*), *gc*, *cp*, and *bhmt* were barely or weakly expressed in the regenerating liver of the *khdrbs1*^−/−^ mutant at R48 h (Fig. 3E). Additionally, Hematoxylin and Eosin (H&E) staining revealed an atypical structure of regenerating liver in *khdrbs1*^−/−^ mutant at R48 h compared to the control (Fig. S5G). Taken together, these data indicate that Khdrbs1 is indispensable for biliary-mediated liver regeneration.

### *khdrbs1* is required for the re-differentiation and proliferation of BPPCs during liver regeneration

After hepatocyte ablation, BECs can contribute to liver regeneration through de-differentiation and re-differentiation into hepatocytes (Choi et al., 2014; He et al., 2014). To evaluate the de-differentiation of BECs in *khdrbs1*^−/−^ mutants after liver injury, FISH and qPCR assays showed comparable expression of the hepatoblast markers *sox9b*, *foxa3*, and *hhex* in Anxa4^+^ BPPCs in *khdrbs1*^−/−^ mutants and controls (Fig. S7A-C). These results suggest that BECs of *khdrbs1*^−/−^ mutants are normally de-differentiated after hepatocyte ablation, like the cells in controls. WISH assays revealed that *khdrbs1*^−/−^ mutants had reduced expression of cell proliferation markers and hepatocyte markers at R24 h, including *myca* (*MYC proto-oncogene, bHLH transcription factor a*), *ccnd1* (*cyclin D1*), *hnf4a*, *bhmt*, *tfa* (*transferrin-a*), *apoa2* (*apolipoprotein A-II*), *gc*, and *cp* (Fig. S7D,E). However, WISH assays showed that *myca* and *ccnd1* were barely expressed in the uninjured livers at 5 dpf, and hepatocyte markers were expressed at normal levels in uninjured livers of *khdrbs1*^−/−^ mutants, compared to controls (Fig. S8A,B). These data indicate that the re-differentiation process of BPPCs is impaired in *khdrbs1*^−/−^ mutants. To assess the re-differentiation process of BPPCs into hepatocytes further, we validated the expression of the biliary marker Alcam and Hnf4α or Dendra2 at R48 h. Alcam-expressing cells co-expressing Hnf4α or Dendra2 were more abundant in *khdrbs1*^−/−^ mutants at R48 h, compared to controls (Fig. 3F,G), suggesting that the re-differentiation of BPPCs into hepatocytes is impaired after Khdrbs1 deficiency during liver regeneration.

The proliferation of liver progenitor cells is essential for re-differentiation during liver regeneration (So et al., 2020). Based on this, we used 5-ethynyl-2′-deoxyuridine (EdU) to label proliferating BPPCs at R8 h (Fig. S9A). There were fewer EdU^+^ cells among Anxa4^+^ BPPCs in *khdrbs1*^−/−^ mutants at R8 h than the control. At R24 h, the number of EdU and Dendra2 double-stained BPPCs in *khdrbs1*^−/−^ mutants was also less than the control (Fig. S9B-E), indicating poor proliferation of BPPCs in *khdrbs1*^−/−^ mutants during liver regeneration. However, consistent with the control, the TUNEL (terminal deoxynucleotidyl transferase biotin-dUTP nick end labeling) assay could not detect apoptotic signals in Dendra2^+^ BPPCs of *khdrbs1*^−/−^ mutants at R24 h (Fig. S9F). Furthermore, the apoptotic cell marker cleaved Caspase3 (CL-Caspase3) was also not detected in BPPCs of *khdrbs1*^−/−^ mutants, but a low survival rate was observed after R48 h (Fig. S9G,H). These data suggest that loss of Khdrbs1 hinders the proliferation of BPPCs and does not cause apoptosis in biliary-mediated liver regeneration.

### Activated p53 signaling responds to the loss of Khdrbs1 and impairs the re-differentiation of BPPCs during liver regeneration

**Fig. 4.**
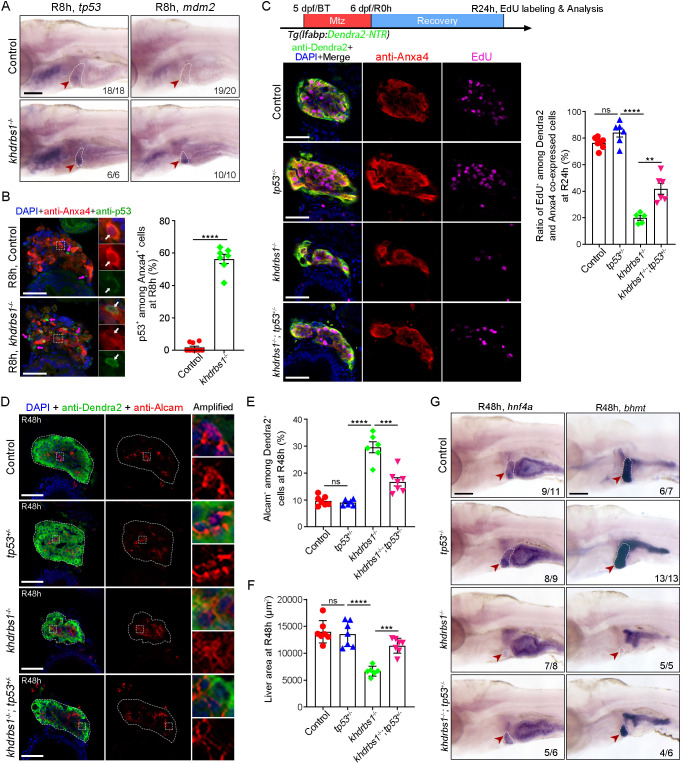
**p53 mutation partially rescues liver regeneration in the *khdrbs1* mutant.** (A) WISH images showing the expression of *tp53* and *mdm2* in the control and *khdrbs1*^−/−^ mutant at R8 h. (B) Confocal images and quantification showing the expression of p53 in Anxa4^+^ cells at R8 h. Insets show magnified views of regions of interest. White arrows point to p53 and Anxa4 co-expressing cells, and magenta arrows point to p53^+^ and Anxa4^−^ cells. Control, *n*=9; *khdrbs1*^−/−^, *n*=7. (C) EdU assay (schematic) showing the proliferation of BPPCs after *tp53* mutation R24 h. Graph shows quantification of EdU^+^ among Dendra2 and Anxa4 co-expressing cells. Control, *n*=7; *tp53*^−/−^, *n*=6; *khdrbs1*^−/−^, *n*=5; *khdrbs1*^−/−^*;tp53*^+/−^, *n*=6. (D) Confocal images showing the expression of Alcam and Dendra2 at R48 h. Insets show magnified views of regions of interest. Dashed lines delineate liver region. (E,F) Quantification of Alcam^+^ cells among Dendra2^+^ cells (E) or the area of regenerating livers (F) after *tp53* mutation at R48 h. Control, *n*=7; *tp53*^−/−^, *n*=7; *khdrbs1*^−/−^, *n*=6; *khdrbs1*^−/−^*;tp53*^+/−^, *n*=7. (G) WISH images showing the expression of *hnf4a* and *bhmt* after *tp53* mutation at R48 h. Scale bars: 100 μm. In WISH images, red arrowheads and white dashed outlines indicate livers, and numbers indicate the proportion of larvae exhibiting the expression shown. ns, not significant. **P<0.01, ***P<0.001, ****P<0.0001 (two-tailed, unpaired *t*-test). Error bars represent s.e.m. Scale bars: 100 μm (A); 50 μm (B-D).

KHDRBS1 has been reported to function as a co-activator of p53 (Li and Richard, 2016). Moreover, our previous research revealed negative effects of activated p53 during liver regeneration (He et al., 2022). Given the different observations and impaired liver regeneration after Khdrbs1 loss, we then examined the expression patterns of p53-associated genes (*tp53*, *mdm2*, *cdkn1a*, and *ccng1*) (Chen et al., 2005; Okamoto and Beach, 1994). Our single-cell RNA-sequencing data from wild-type livers showed the expression of p53-associated genes in BPPCs at R0 h, with *tp53* and *mdm2* lower in abundance (Fig. S10A). In comparison to the control, WISH analysis showed significant upregulated expression of *tp53*, *mdm2*, and *ccng1* across broad anatomical regions in *khdrbs1*^−/−^ mutants, including the optic tectum, pharyngeal pouches, and gut, but not in the liver at 3.5 dpf and 5 dpf (Fig. S10B,C). Further investigations using WISH indicated that *tp53* and *mdm2* expression was significantly upregulated in the regenerating livers of *khdrbs1*^−/−^ mutants and the dCas9i group at R8 h (Fig. 4A; Fig. S11A,B). FISH and antibody assays revealed that p53 was upregulated in BPPCs of *khdrbs1*^−/−^ mutants at R8 h (Fig. 4B; Fig. S11C). Moreover, the expression levels of *tp53*, *mdm2*, and *ccng1* were significantly increased in the regenerating livers of *khdrbs1*^−/−^ mutants at R24 h (Fig. S10D). Notably, Δ*113tp53* is a variant of *tp53* produced by an alternative internal promoter in the *tp53* gene and acts as a target gene of p53 in zebrafish (Guo et al., 2010). Given that the *tp53* RNA probe used in WISH could not distinguish between Δ*113tp53* and full-length of *tp53* (*tp53-FL*), qPCR was performed to further evaluate p53-associated genes. qPCR results substantiated the upregulated expression of p53 target genes, including Δ*113p53*, *mdm2*, and *ccng1*, but not *tp53-FL* and *cdkn1a*, in the *khdrbs1*^−/−^ mutant at R24 h (Fig. S10E), implying that p53 activity was induced and weakly dependent on the transcription of *tp53-FL* after loss of Khdrbs1. WISH of *tp53* expression was also increased in dCas9i group at R24 h after Dox treatment (Fig. S10F). Therefore, these results suggest another interaction between Khdrbs1 and p53 during biliary-mediated liver regeneration that does not involve reinforcement of p53 signaling.

To confirm that impaired liver regeneration caused by Khdrbs1 deficiency is due to activated p53 signaling, we introduced *tp53^M214K^* into the *khdrbs1*^−/−^ mutant, in which *tp53^M214K^* encoded an inactive p53 (Berghmans et al., 2005). As a result, the proliferation rate of BPPCs was partially rescued by heterozygous *tp53* (*tp53^+/−^*) in *khdrbs1*^−/−^ mutants at R24 h, but no significant rescue was observed at R8 h (Fig. 4C; Fig. S11D). Additionally, the proportion of Alcam^+^ cells among Dendra2^+^ cells was diminished by *tp53* heterozygous mutation in *khdrbs1*^−/−^ mutants (Fig. 4D,E), indicating the rescued re-differentiation of BPPCs. Moreover, the regenerating liver size of *khdrbs1*^−/−^ mutants was also partially rescued at R48 h (Fig. 4F), as well as the expression of hepatocyte markers *hnf4a* and *bhmt* (Fig. 4G). Furthermore, *tp53* homozygous mutation in *khdrbs1*^−/−^ mutants also partially rescued the re-differentiation and regenerating liver size of *khdrbs1*^−/−^ mutants at R48 h (Fig. S12A-D). These data suggest that p53 is a downstream effector of Khdrbs1 in biliary-mediated liver regeneration.

p53 is an essential transcription factor involved in tumorigenesis and tissue repair. Aberrant activation of p53 has been implicated in blocking hepatocyte proliferation and identity (Ji et al., 2017; Munroe et al., 2020). Our previous work showed that activated p53 impaired the initiation of biliary-mediated liver regeneration, re-differentiation of BPPCs, and survival of nascent hepatocytes in zebrafish (He et al., 2022; Song et al., 2023). Activated p53 in mice acts as a ‘protector’ in hepatotoxicity-induced liver damage by enhancing antioxidative capacity (Xu et al., 2022; Zhou et al., 2021). Our study revealed that p53 accumulation is triggered by the loss of Khdrbs1 in BPPCs during liver regeneration, hinting that Khdrbs1 may inhibit the p53 activity rather than function as a co-activator of p53 (Li and Richard, 2016). In conclusion, our study demonstrates that Khdrbs1 is an essential factor in boosting the re-differentiation of BPPCs into hepatocytes after severe liver injury by the spatiotemporal knockdown method and genetic mutation. p53 signaling, which is specifically activated in response to the loss of Khdrbs1 after liver injury, acts as an obstructive effector for biliary-mediated liver regeneration. These findings highlight a previously unrecognized role of Khdrbs1 in liver regeneration and provide further insight into the regulation of biliary-mediated liver regeneration.

## MATERIALS AND METHODS

### Zebrafish strains

Zebrafish were maintained under the Guidelines of the Institutional Animal Care and Use Committee protocols from Southwest University (2007). All experimental procedures were performed following the standard guidelines and approved by the Institute of Developmental Biology and Regenerative Medicine, Southwest University (Chongqing, China).

Zebrafish strains used were: *Tg(lfabp:Dendra2-NTR)^cq1^*; *Tg(krt18:CreER)^cq74^*; *Tg(lfabp:loxp-stop-loxp-DsRed)^cq4^*; *Tg(tp1bglob:eGFP)^um14^*, abbreviated as *Tg(Tp1:GFP)*; *TgBAC(hand2:GFP)^cq106^*; *tp53^M214K^*; *Tg(krt18:Tet-on 3G)^cq157^*; *Tg(TRE3G:dCas9NLS-T2A-GFP;U6:gRNA-khdrbs1a;cryaa:venus)^cq218^*; *khdrbs1a^cq219^*; and *khdrbs1b^cq220^*. Embryos were incubated with 0.003% 1-phenyl-2-thiourea (Sigma-Aldrich) from 24 hpf to inhibit pigmentation.

### Drug treatment

To induce ablation of hepatocytes, *Tg(lfabp:Dendra2-NTR)* larvae were incubated for 21 h with 12 mM Mtz (Sangon) in 0.2% DMSO at 5 dpf. After incubation with Mtz, larvae were washed three times with egg water and then re-covered in egg water at 28.5°C. *Tg(TRE3G:dCas9NLS-T2A-GFP;U6:gRNA-khdrbs1a;cryaa:venus*; *krt18:Tet-on 3G)* and *Tg(TRE3G:dCas9NLS-T2A-GFP;U6:gRNA-khdrbs1a;cryaa:venus)* were treated with Dox (20 µg/ml; Sangon) at 4 dpf. For lineage tracing during liver regeneration, *Tg(lfabp:loxp-stop-loxp-DsRed*;*krt18:CreER)* were incubated with DMSO or 4-hydroxytamoxifen (5 µM; Sigma-Aldrich) from 4 dpf to 5 dpf.

### Generation and genotyping of *khdrbs1a* and *khdrbs1b* mutants

The CRISPR/Cas9 method was used to generate *khdrbs1a^cq219^* and *khdrbs1b^cq220^* lines. The target sequences of *khdrbs1a* gRNAs, 5′-GAAGCACCGACCTGCAGATAGG-3′ and 5′- GGCCTGTTGGTGGGAGAGTCCGG-3′, are respectively located in exon 1 and exon 6 of the *khdrbs1a* genome (PAM site underlined). The target sequence of *khdrbs1b* gRNA 5′- GATCTCCGTGCTGGGAAAAGG-3′ is located in exon 3 of the *khdrbs1b* genome (PAM site underlined). gRNAs were separately synthesized following the descriptions (Chang et al., 2013). Cas9 protein (20 µM, NEB) or *Cas9* mRNA (100 ng/µl) and gRNA(s) (150 ng/µl) were mixed and then co-injected into one-cell-stage, wild-type embryos. Genotyping was performed according to previous descriptions (He et al., 2019). Primers for PCR and sequencing were: *khdrbs1a*-exon1 forward, 5′-GGATACGTCACTTCCATCTG-3′; *khdrbs1a*-exon1 reverse, 5′-CCAAACCACAATCACTGCACGC-3′; *khdrbs1a*-exon6 forward, 5′-GTTGTCTTTAATAGGGGTCGTG-3′; *khdrbs1a*-exon6 reverse, 5′-GCATTGGGGCTGAATAAACAAT-3′; *khdrbs1b*-exon3 forward, 5′- GTTAAGTGAAGCACTGTAGG-3′; *khdrbs1b*-exon3 reverse, 5′-GGTGCCCCGGGAAATATTG-3′.

### Generation of *Tg(TRE3G:dCas9NLS-T2A-GFP;U6:gRNA-khdrbs1a; cryaa: venus)*

The plasmid *pTol2-TRE3G:dCas9NLS-T2A-GFP;U6:gRNA-khdrbs1a;cryaa:venus* was generated by replacing the Cas9 coding sequence (CDS) with a dead Cas9 CDS fragment. The U6-gRNA-khdrbs1a fragment was cloned from a U6 fragment and gRNA-khdrbs1a fragment by overlap PCR, digested with SacII and NheI. The dead Cas9 CDS was cloned by site-directed mutagenesis from the Cas9 CDS. Vector plasmid and dead Cas9 CDS fragment were digested by NcoI and DraIII and then ligation was performed for mutation of D10A as the first step. Second, a fragment of H839A and new vector generated from the first step were digested by EcoRI followed by ligation. *Tg(TRE3G:dCas9NLS-T2A-GFP;U6:gRNA-khdrbs1a;cryaa:venus)* was generated by injection of the plasmid *pTol2-TRE3G:dCas9NLS-T2A-GFP;U6:gRNA-khdrbs1a;cryaa:venus* (15 ng/µl) and *Tol2* mRNA (50 ng/µl) into one-cell-stage embryos.

### WISH and FISH

WISH experiments and FISH coupled with antibody staining assays were performed as previously described (He et al., 2019). All larvae were fixed with 4% paraformaldehyde in PBS at 4°C overnight and then dehydrated and incubated in 100% methanol at −30°C for at least 24 h. Respective cDNAs from whole embryos at 48 hpf and 5 dpf were used to amplify the genes. PCR products served as templates for the synthesis of the probes using a digoxigenin RNA labeling kit (Roche Applied Science). WISH embryos were imaged with a Zeiss microscope (SteREO DiscoveryV20) equipped with Axio Vision Rel 4.8.2 software. FISH larvae were imaged with an LSM780 confocal microscope (Carl Zeiss) equipped with ZEN2010 software. Table S1 lists the details of probe primers.

### Antibody staining and H&E staining

Antibody staining was performed as previously described (He et al., 2014). DAPI (4′,6-diamidino-2-phenylindole; 1:5000; Sigma-Aldrich, D8417) was used to stain nuclei. Primary antibodies used were: rabbit anti-Dendra2 (1:1000; Evrogen, AB821), goat anti-GFP (1:1000; Abcam, ab6658), mouse anti-Anxa4 (1:500; 2F11, Abcam, ab71286), goat anti-Hnf4α (1:50; Santa Cruz Biotechnology, sc6556), mouse anti-Alcam (1:50; ZIRC, zn-5), rabbit anti-cleaved Caspase3 (1:200; Cell Signaling Technology, 9664S), mouse anti-DsRed (1:500; Santa Cruz Biotechnology, sc101526), rabbit anti-Lcp1 (1:400; Li et al., 2011), and rabbit anti-p53 (1:500; GeneTex, GTX128135). Secondary antibodies were: Alexa Fluor 488-conjugated donkey anti-rabbit IgG (1:1000; Invitrogen, A21206), Alexa Fluor 568-conjugated donkey anti-mouse IgG (1:1000; Invitrogen, A10037), Alexa Fluor 633-conjugated donkey anti-goat IgG (1:1000; Invitrogen, A21082), Alexa Fluor 647-conjugated donkey anti-mouse IgG (1:1000; Invitrogen, A31571), and Alexa Fluor 647-conjugated donkey anti-rabbit IgG (1:1000; Invitrogen, A31573). Images were photographed using an LSM780 confocal microscope (Carl Zeiss) equipped with ZEN2010 software.

H&E staining was performed according to a standard protocol. Samples were cut into 7-μm-thick sections, which were stained using H&E to examine tissue morphology. H&E images were taken using a Zeiss Axio Imager Z1 microscope (Carl Zeiss).

### EdU labeling and TUNEL assay

The EdU and TUNEL assays were performed as previously described (He et al., 2014). EdU (200 µM) was injected into the heart at R8 h or R24 h. After recovery for 40-60 min, injected larvae were fixed in 4% paraformaldehyde.

### Single-cell RNA sequencing and qPCR analysis

Single-cell RNA-sequencing data were analyzed and uniform manifold approximation and projection (UMAP) graphs generated using the Loupe Browser (version 5.0.1) (Cai et al., 2021). qPCR was performed using the FastStart Universal SYBR Green Master kit (Roche). To evaluate the expression of *khdrbs1a* and *khdrbs1b*, total RNA was extracted from 30-50 livers stripped with fine forceps in PBS from wild-type embryos at 6 dpf, R0 h, and R8 h using Tripure isolation reagent (Roche). To evaluate the expression of hepatoblast markers and p53-associated genes, total RNA was extracted from 25 stripped livers from the control or *khdrbs1*^−/−^ mutant at R8 h and R24 h. cDNA was synthesized using the Omniscript RT Kit (QIAGEN) according to the manufacturer's protocol. Table S2 lists qPCR primers.

### Quantification of liver size and statistical analysis

The area (µm^2^) of liver and intensity of fluorescence were measured with ZEN 3.0 software (Carl Zeiss). ImageJ version 1.46 was used to quantify the number of cells. All statistical tests were performed with GraphPad Prism version 9.0 for Windows (GraphPad Software). The data were analyzed with two-tailed, unpaired *t*-test, and multiple comparisons performed with analysis of variance tests were used to determine statistical significance. Quantitative data are presented as mean±s.e.m. Statistical significance was defined as *P<0.05, **P<0.01, ***P<0.001, and ****P<0.0001.

## Supplementary Material



10.1242/develop.204266_sup1 Supplementary information

Table S1. Primer sequences used for probe synthesis in WISH and FISH

Table S2. Primer sequences used for qPCR
