# Supplementary material for: Khdrbs1 drives re-differentiation of bipotential progenitor cells by inhibiting p53 in zebrafish biliary-mediated liver regeneration
Source: Development. 2025 Feb 28;152(4):DEV204266. doi: 10.1242/dev.204266 (PMC11928054; doi:10.1242/dev.204266)
Supplement: Supplementary information [file develop-152-204266-s1.pdf]

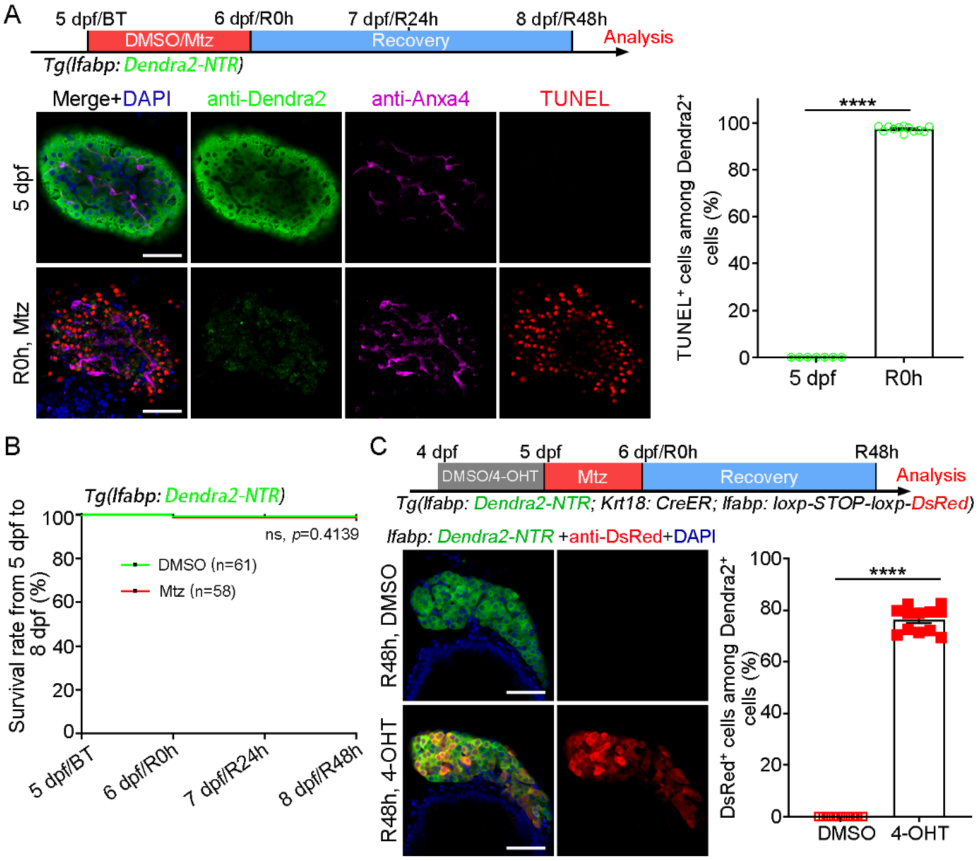

Fig. S1. Hepatocytes loss induces biliary-mediated liver regeneration. (A) Scheme showing the stages of DMSO/Mtz treatment and analysis during liver regeneration. Confocal images showing TUNEL$^+$ cells, Dendra2$^+$ hepatocytes and Anxa4$^+$ BECs at 5dpf (BT, before Mtz treatment) and R0h after DMSO/Mtz treatment. Quantification of TUNEL and Dendra2 merged hepatocytes. (B) Survival curves of DMSO/Mtz treated larvae from 5dpf to R48h. (C) *Tg(krt18:CreER;lfabp:loxp-STOP-loxp-DsRed)* was used to label BECs after DMSO/4-OHT (4-hydroxytamoxifen) treatment and analyzed at R48h. Confocal images showing the labeled BECs contribute to hepatocyte after 4-OHT treatment. Quantification of the DsRed$^+$ among Dendra2$^+$ cells. ns: not significant. Asterisks indicate statistical significance: ****$p < 0.0001$. Error bars, ±SEM. Scale bar: 50 μm.

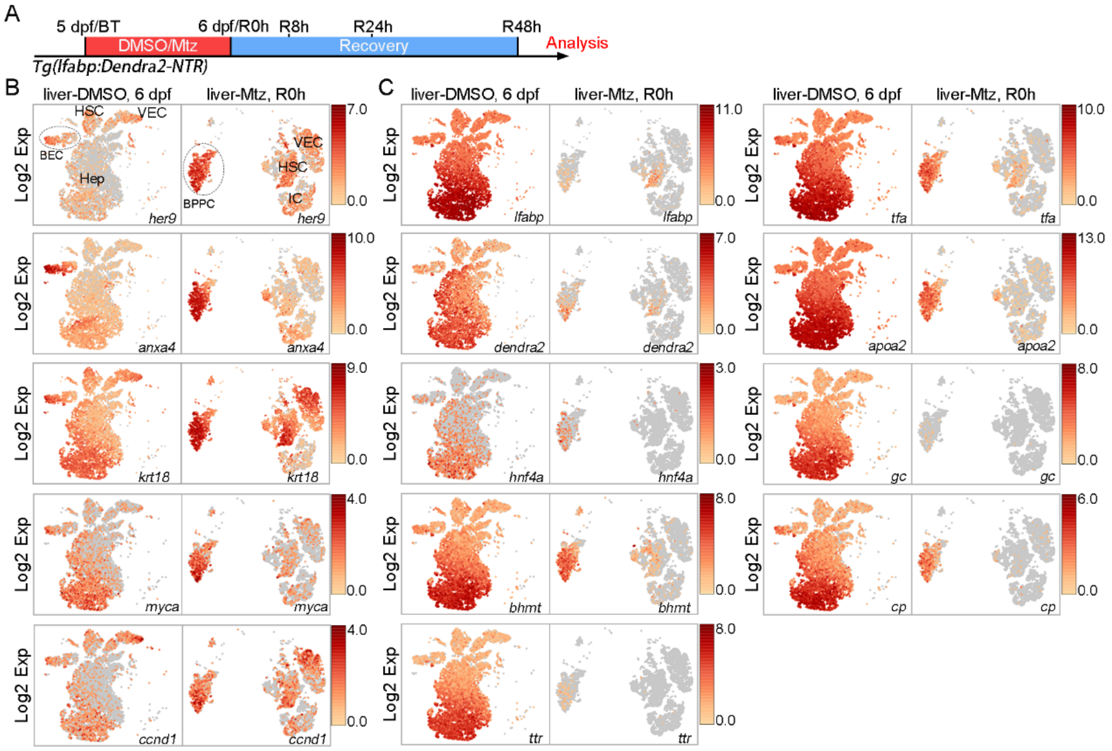

Fig. S2. The single-cell RNA sequencing data of BEC/BPPC markers, cell proliferation markers, and hepatocyte markers. (A) Scheme showing the stages of DMSO/Mtz treatment and analysis during liver regeneration. (B-C) Uniform manifold approximation and projection (UMAP) showing the expression of BEC/BPPC markers *her9* (hairy-related 9), *anxa4* (annexin A4), and *krt18* (keratin 18a, tandem duplicate 1), cell proliferation markers *myca* (MYC proto-oncogene, bHLH transcription factor a) and *ccnd1* (cyclin D1), and hepatocyte markers *lfabp* (fatty acid binding protein 10a), *Dendra2*, *hnf4a* (hepatocyte nuclear factor 4 alpha), *bhmt* (betaine-homocysteine methyltransferase), *ttr* (transthyretin), *tfa* (transferrin-a), *gc* (GC vitamin D binding protein), and *cp* (ceruloplasmin) (overlayed red) in livers of wide-type at 6 dpf and R0h, respectively. Hep, hepatocyte; BEC, biliary epithelial cell; BPPC, bipotential progenitor cell; HSC, hepatic stellate cell; VEC, vascular endothelial cell; IC, immune cell. Black dashed circle indicates BEC/BPPC.

Development • Supplementary information

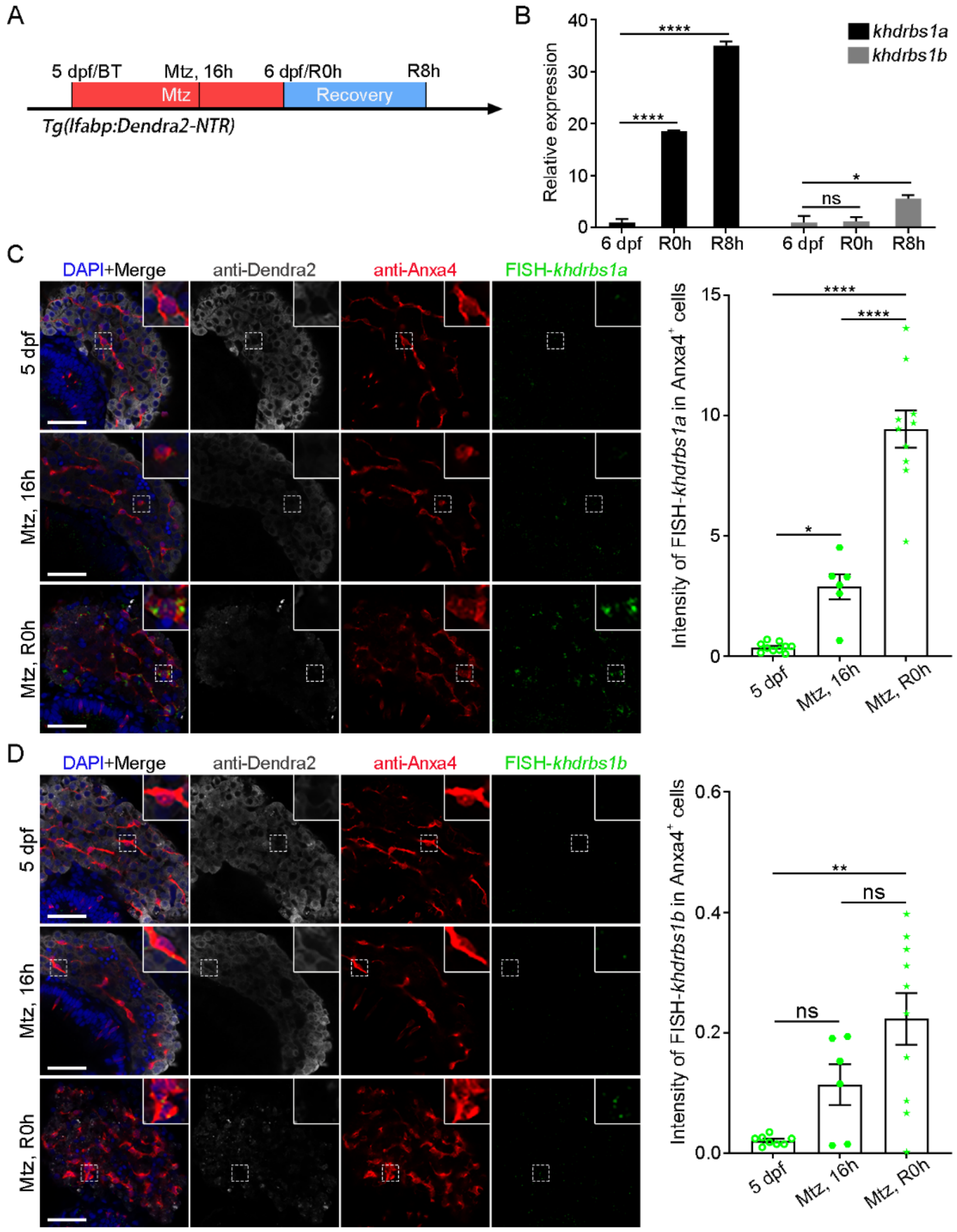

Fig. S3. *khdrbs1a* and *khdrbs1b* are expressed in BPPCs at R0h during liver regeneration. (A) Scheme showing the stages of Mtz treatment and analysis during liver regeneration. (B) qPCR analysis showing the expression levels of *khdrbs1a* and *khdrbs1b* at 6 dpf, R0h, and R8h. (C-D) Confocal images showing the expressions of *khdrbs1a* or *khdrbs1b* in Anax4$^+$ BPPCs at 5 dpf, Mtz-16h, and R0h via FISH, respectively. Quantification of the intensity of FISH-*khdrbs1a* and FISH-*khdrbs1b* expression in Anax4$^+$ BPPCs. Scale bar: 50 μm. ns: not significant. Asterisks indicate statistical significance: *$p < 0.05$, **$p < 0.01$, ****$p < 0.0001$. Error bars, ±SEM. Scale bar: 50 μm.

Development • Supplementary information

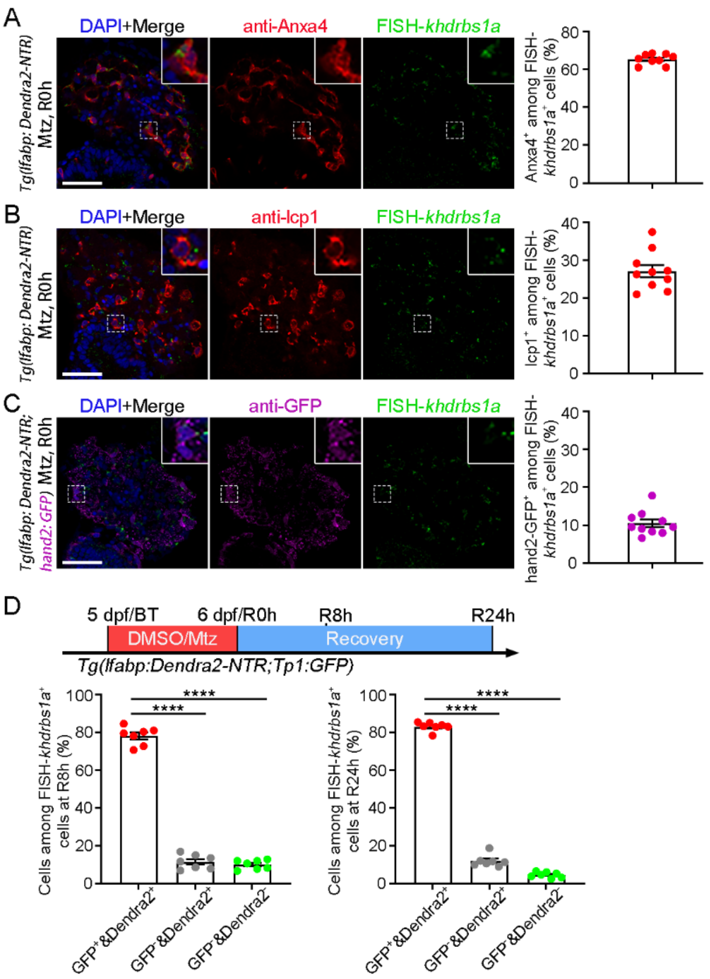

Fig. S4. The expression patterns of *khdrbs1a* in BPPCs and other cells. (A-C) Confocal images showing the expressions of *khdrbs1a* via FISH in Anax4⁺ BPPCs, Icp1⁺ macrophages, or hand2-GFP⁺ hepatic stellate cells at R0h. Quantification of the Anxa4⁺, Icp1⁺, or hand2-GFP⁺ cells among FISH-*khdrbs1a* positive cells at R0h. (D) The cells expressing FISH-*khdrbs1a* at R8h and R24h. *Tg(lfabp:Dendra2-NTR; Tp1:GFP)* labeling hepatocytes and BECs, respectively. Asterisks indicate statistical significance: ****p < 0.0001. Error bars, ±SEM. Scale bar: 50 μm.

Development • Supplementary information

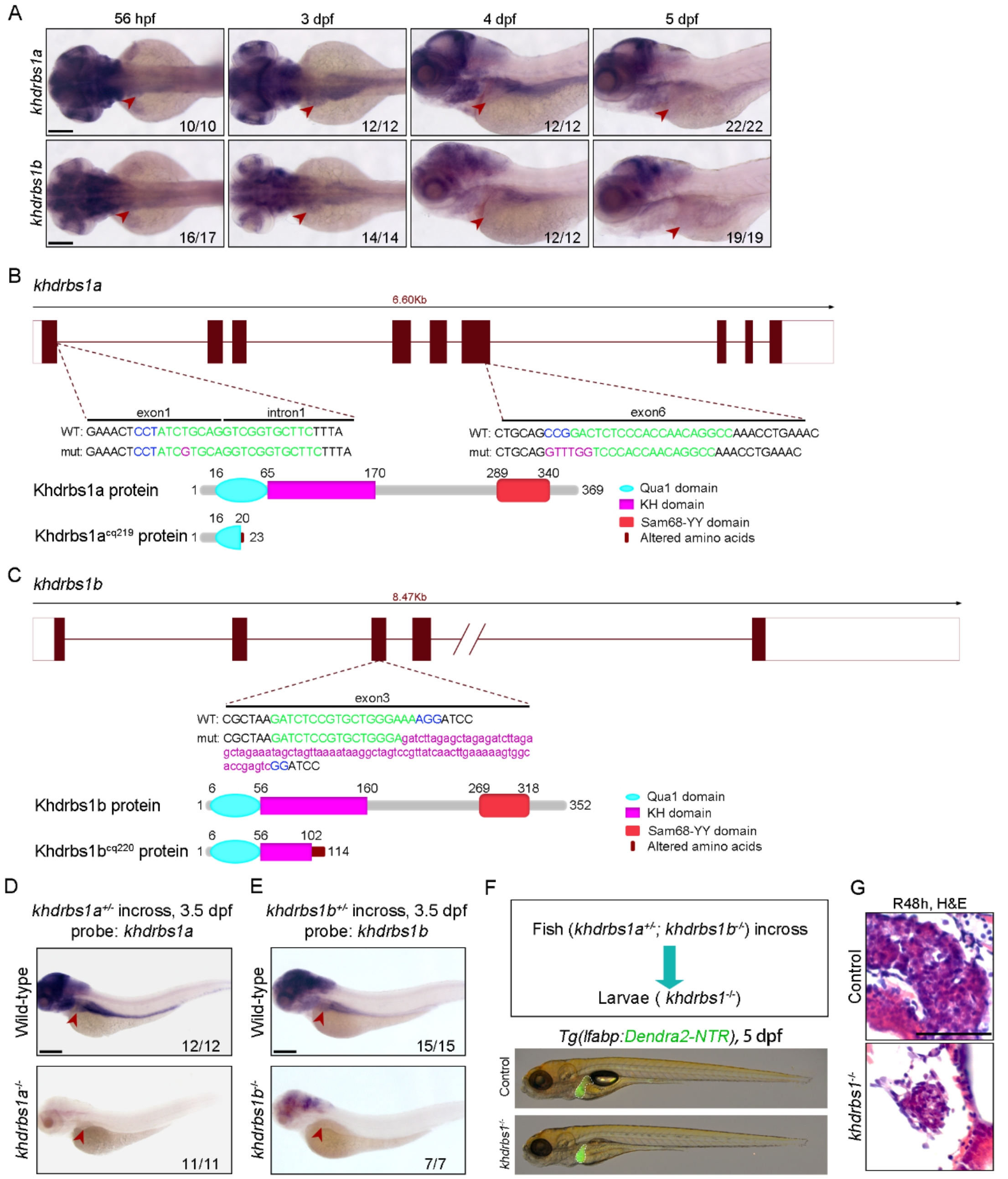

Fig. S5. Genetic deletion of *khdrbs1a* and *khdrbs1b* via CRISPR/Cas9 method. (A) WISH images showing the expression of *khdrbs1a* or *khdrbs1b* in the wide-type at 56 hours post fertilization (hpf), 3 dpf, 4 dpf, and 5 dpf. Scale bar: 100 μm. (B-C) Schemes showing gRNA targets and sequences (green) of *khdrbs1a* and *khdrbs1b*, respectively. PAM (protospacer adjacent motif), marked with blue. Insertion and Indel, marked with purple. Protein schemes showing the structure of the expected proteins. (D-E) WISH images of *khdrbs1a* or *khdrbs1b* in wide-type and its mutant at 3.5 dpf, Scale bar: 100 μm. (F) The

generated strategy of *khdrbs1*$^{-/-}$ *mutant*. (G) H&E staining of the regenerating livers of control and *khdrbs1*$^{-/-}$ *mutant* at R48h, Scale bar: 50 μm. Red arrowheads point to livers. Numbers indicate the proportion of larvae exhibiting the expression shown.

Development • Supplementary information

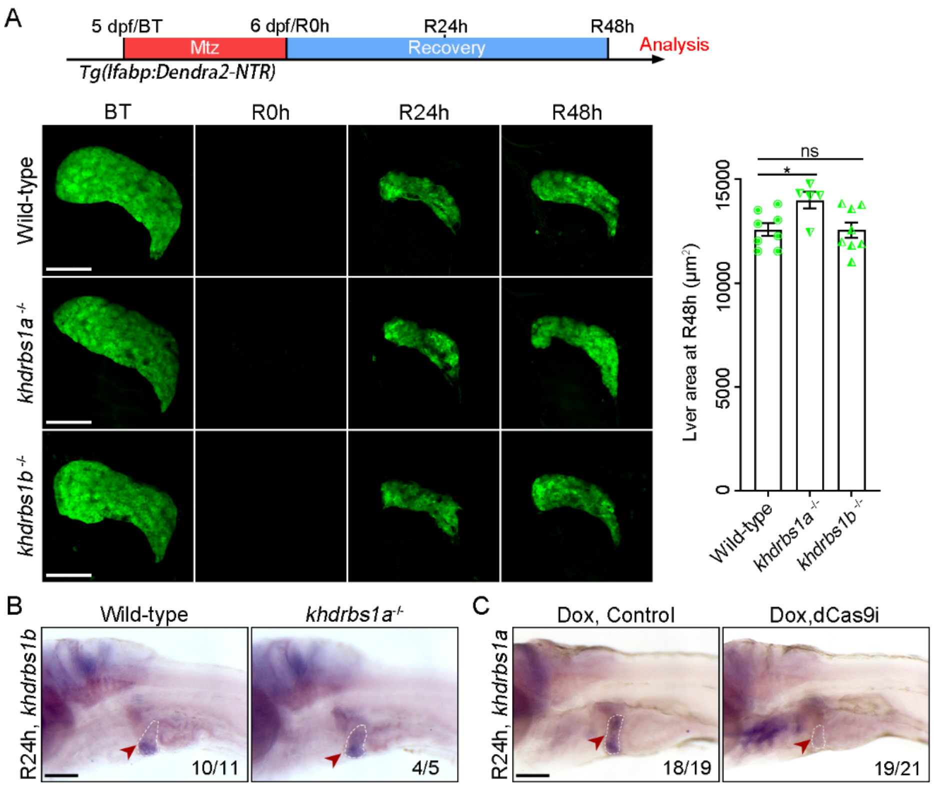

Fig. S6. *khdrbs1a*$^{-/-}$ mutant or *khdrbs1b*$^{-/-}$ mutant displays normal liver regeneration. (A) Images showing the liver regeneration of wild-type, *khdrbs1a*$^{-/-}$ mutant and *khdrbs1b*$^{-/-}$ mutant from BT to R48h, respectively. Quantification of liver area at R48h. (B) WISH images showing the expression pattern of *khdrbs1b* in the *khdrbs1a*$^{-/-}$ mutant at R24h. (C) WISH images showing the expression patterns of *khdrbs1a* in the control and dCas9i group at R24h. Red arrowheads and white-dashed circles point to livers. Numbers indicate the proportion of larvae exhibiting the expression shown. ns: not significant. Asterisks indicate statistical significance: *$p < 0.05$. Error bars, ±SEM. Scale bar: 100 μm.

Development • Supplementary information

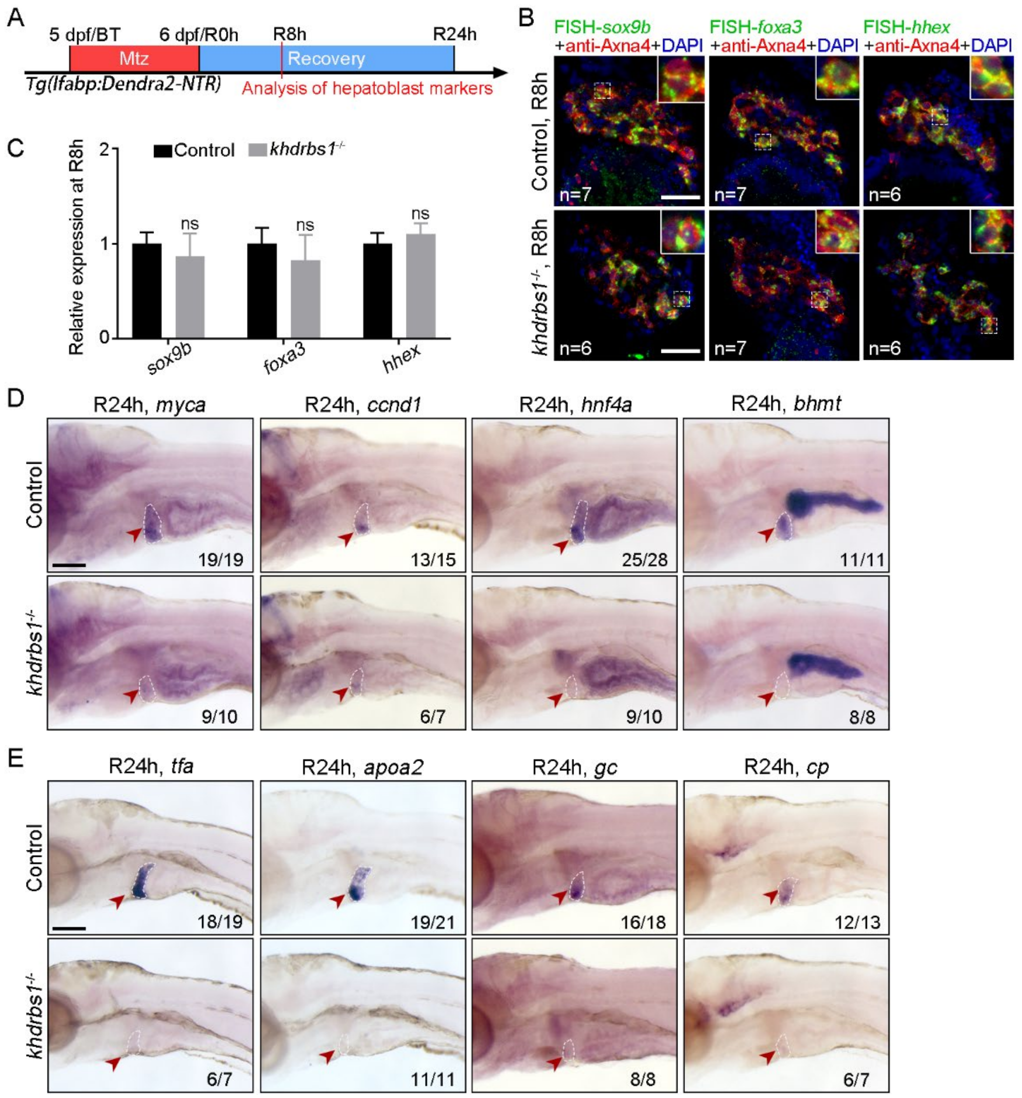

Fig. S7. The dedifferentiation of BECs is normal in *khdrbs1*$^{-/-}$ mutant after hepatocyte ablation but the redifferentiation is stuck. (A) Scheme showing the stage of Mtz treatment and analysis of BPPCs markers in the control and *khdrbs1*$^{-/-}$ mutant at R8h. (B) Single-optical section images via FISH (green) showing the expressions of BPPC markers *sox9b*, *foxa3*, and *hhex* in Anxa4^{+} BPPCs (red) of the control and *khdrbs1*$^{-/-}$ mutant at R8h, respectively. Scale bar: 50 μm. (C) qPCR analysis showing the expressions of BPPCs markers at R8h. (D-E) WISH images showing the expression patterns of cell proliferation markers *myca* and *ccnd1*, hepatocyte markers *hnf4a*, *bhmt*, *tfa*, *apoa2*, *gc*, and *cp* at R24h. Scale bar, 100 μm. Red arrowheads and white-dashed circles point to livers. Numbers indicate the proportion of larvae exhibiting the expression shown. n, the technical replicates. ns, not significant. Error bars, ±SEM.

Development • Supplementary information

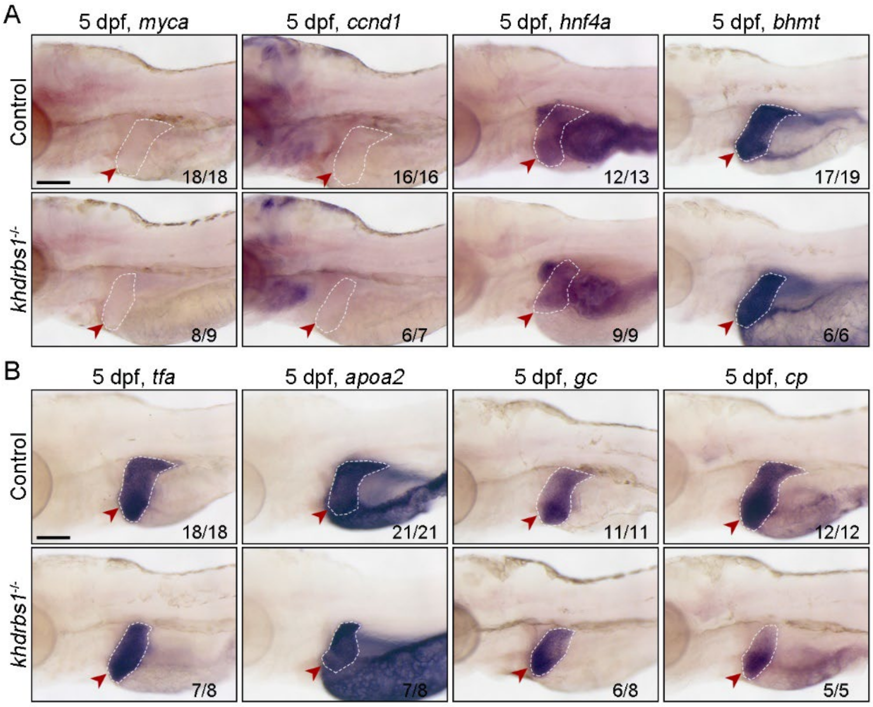

Fig. S8. The expression patterns of liver regeneration markers are comparable in uninjured livers at 5 dpf. (A-B) WISH images showing the expression patterns of cell proliferation markers *myca* and *ccnd1*, hepatocyte markers *hnf4a*, *bhmt*, *tfa*, *apoa2*, *gc*, and *cp* in uninjured livers of the control and *khdrbs1*$^{-/-}$ mutant at 5 dpf. Scale bar, 100 µm. Red arrowheads and white-dashed circles point to livers. Numbers indicate the proportion of larvae exhibiting the expression shown.

Development • Supplementary information

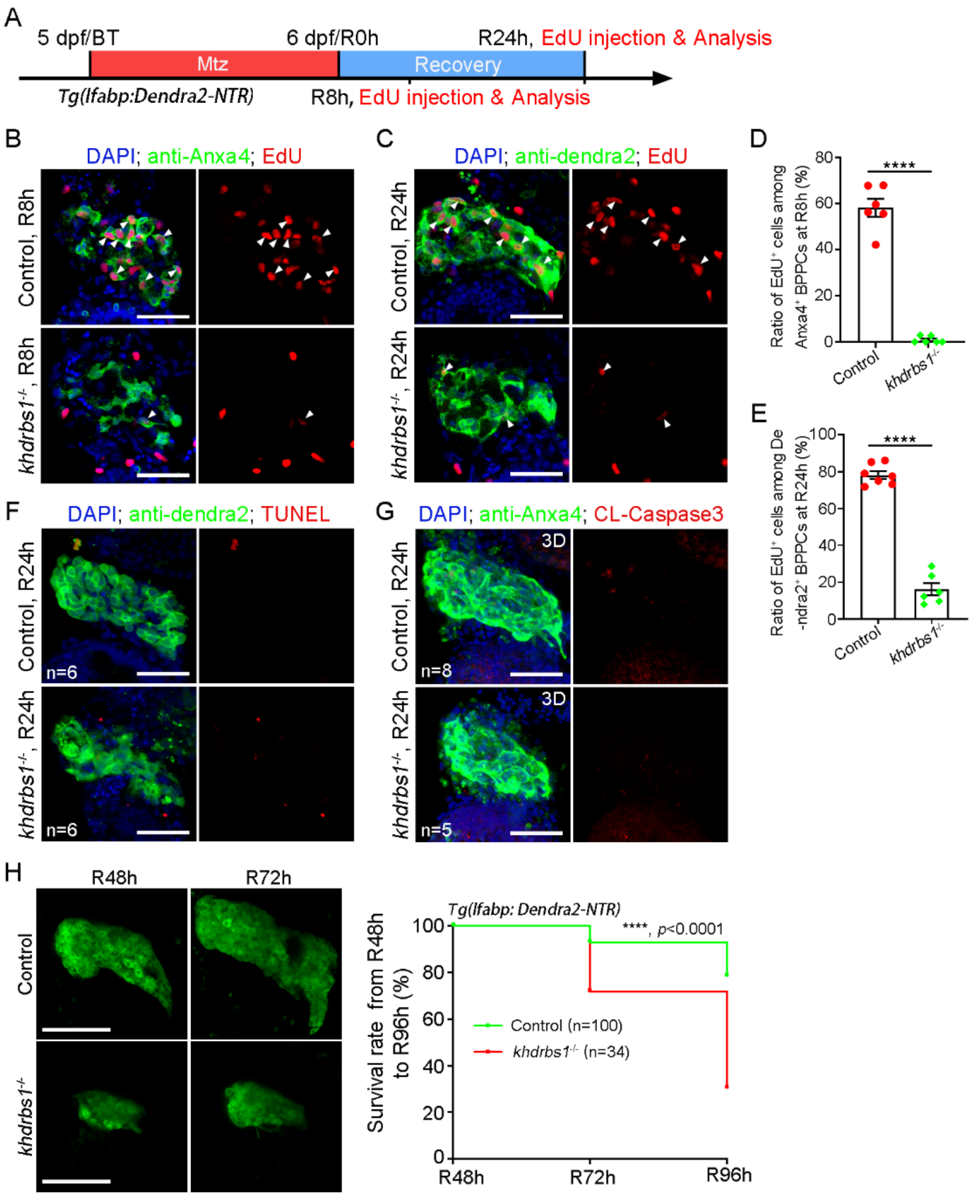

Fig. S9. Loss of *khdrbs1* impedes the proliferation of BPPCs but does not induce apoptosis during liver regeneration. (A) Scheme showing the stages of EdU assay and analysis of BPPCs. (B-C) Images showing the EdU (red) labeling Anxa4^{+} or Dendra2^{+} BPPCs (green) in the control and *khdrbs1*$^{-/-}$ mutant at R8h and R24h, respectively. (D-E) Quantification of the EdU^{+} BPPCs among Anxa4^{+} or Dendra2^{+} BPPCs at R8h and R24h, respectively. (F) Images showing the regenerating livers of TUNEL assay in the control and *khdrbs1*$^{-/-}$ mutant at R24h. (G) Confocal projection images showing antibody assays of CL-caspase3 (cleaved-caspase3) in the control and *khdrbs1*$^{-/-}$ mutant at R24h. (H) Images showing livers of the control and *khdrbs1*$^{-/-}$ mutant at R48h and R72h, Scale bar: 100 μm; Survival curves of the control and *khdrbs1* mutant from R48h to R96h are displayed. Asterisks indicate statistical significance: ****p < 0.0001. Error bars, ±SEM. n, the technical replicates. Scale bar: 50 μm.

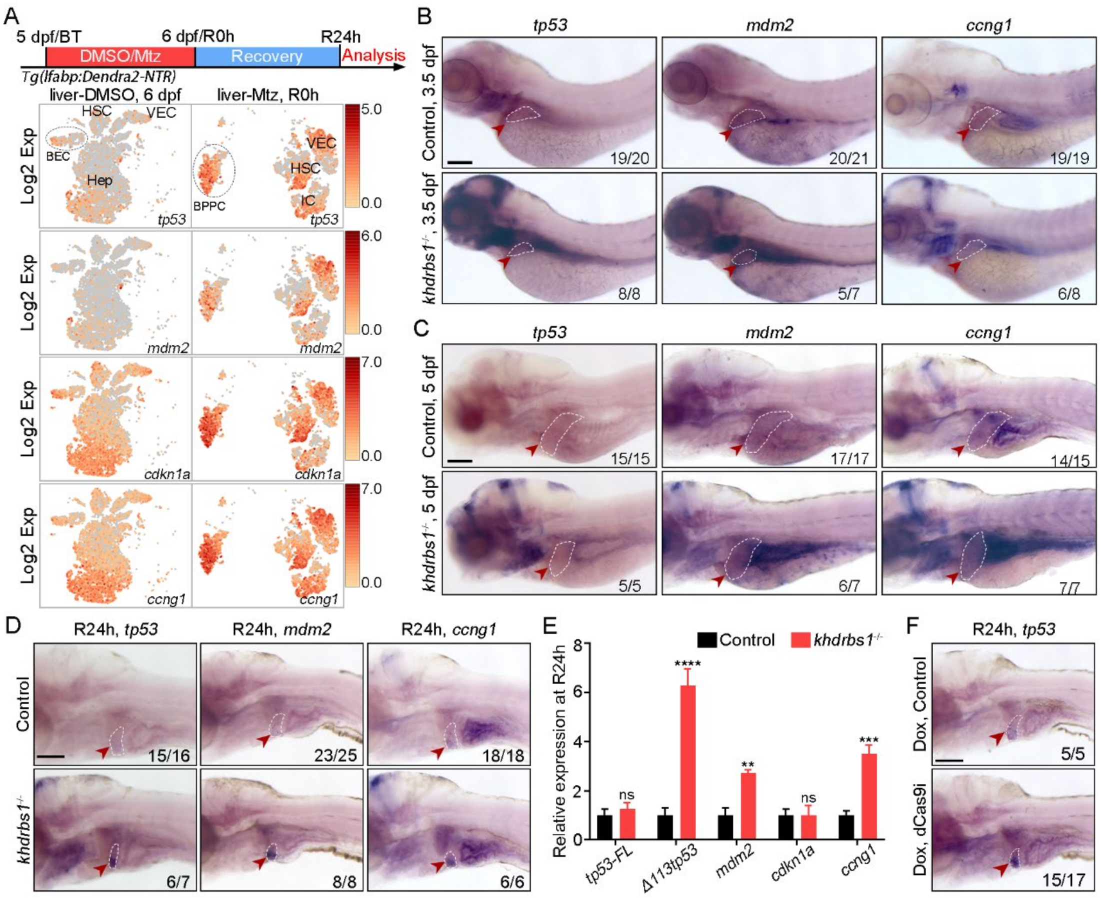

Fig. S10. The activation of p53 occurs in regenerating liver but not undamaged liver of *khdrbs1*$^{-/-}$ mutant. (A) Scheme showing the stages of DMSO/Mtz treatment and analysis during liver regeneration. UMAP showing the basal expression of p53-associated genes in wild-type livers at 6 dpf and R0h. Black dashed circle indicates BEC/BPPC. (B-C) WISH images showing the expression patterns of *tp53*, *mdm2*, and *ccng1* in the control and *khdrbs1*$^{-/-}$ mutant at 3.5 dpf and 5 dpf, respectively. (D) WISH images showing the expression of *tp53*, *mdm2*, and *ccng1* at R24h. (E) qPCR analysis of p53-associated genes including full-length *tp53 (tp53-FL), Δ113tp53, mdm2, cdkn1a,* and *ccng1* at R24h. (F) WISH images showing the expression patterns of *tp53* in the control group and dCas9i group at R24h. Red arrowheads and white-dashed circles point to livers. Numbers indicate the proportion of larvae exhibiting the expression shown. Scale bar: 100 µm. ns: not significant. Asterisks indicate statistical significance: $^{**}p < 0.01$, $^{***}p < 0.001$, $^{****}p < 0.0001$. Error bars, ±SEM.

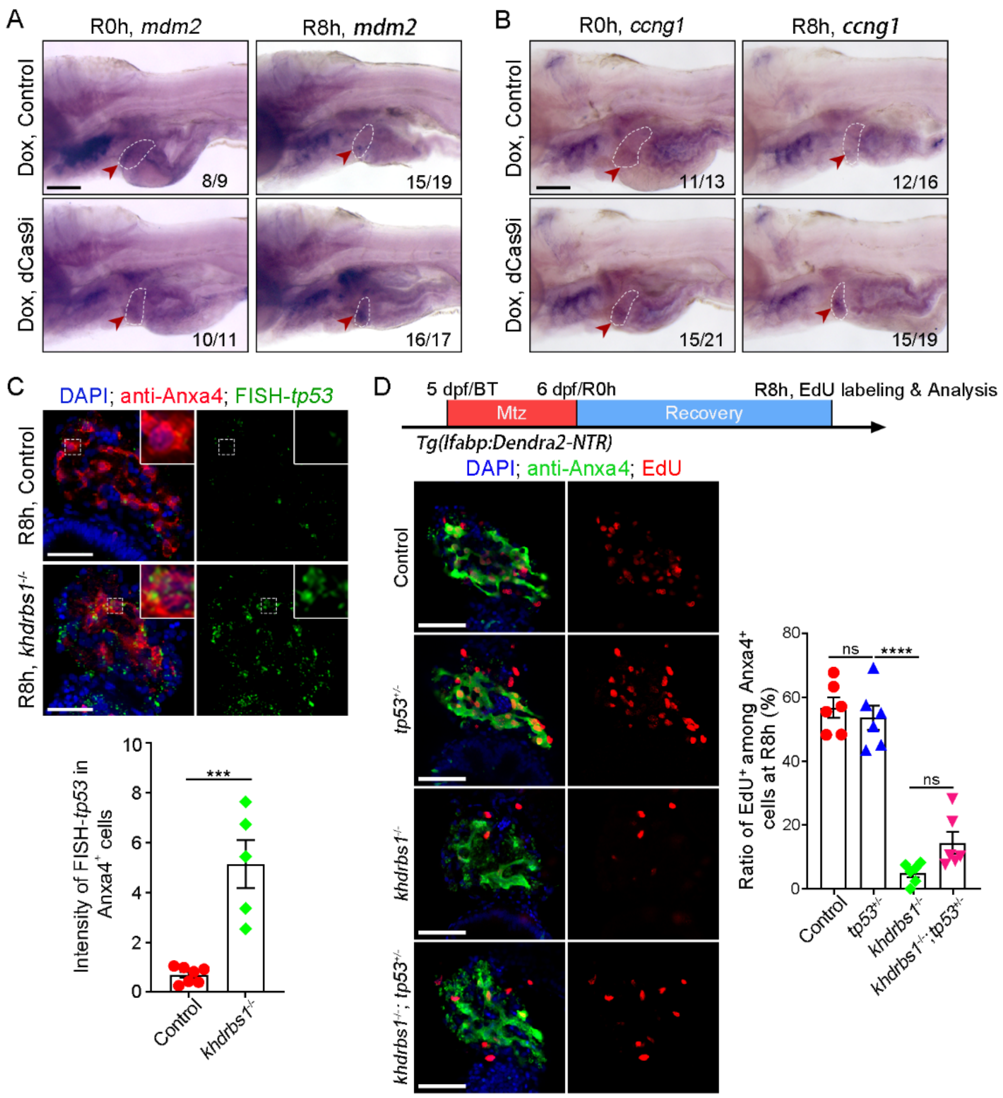

Fig. S11. The activation of p53 occurs in BPPCs of *khdrbs1*$^{-/-}$ mutant. (A-B) WISH images showing the expression patterns of *mdm2* and *ccng1* in the control group and dCas9i group at R0h and R8h, respectively. Scale bar: 100 µm. (C) Images showing the expressions of *tp53* via FISH in Anax4^{+} cells of the control and *khdrbs1*$^{-/-}$ mutant at R8h. Quantification of the intensity of *tp53* expression in Anax4^{+} cells at R8h. (D) EdU Scheme and images showing the proliferation of BPPCs after *tp53* heterozygous mutation at R8h. Quantification of the EdU^{+} BPPCs among Anxa4^{+} BPPCs at R8h. Numbers indicate the proportion of larvae exhibiting the expression shown. Red arrowheads and white-dashed circles point to livers. Scale bar: 50 µm. ns: not significant. Asterisks indicate statistical significance: ***$p < 0.001$, ****$p < 0.0001$. Error bars, ±SEM.

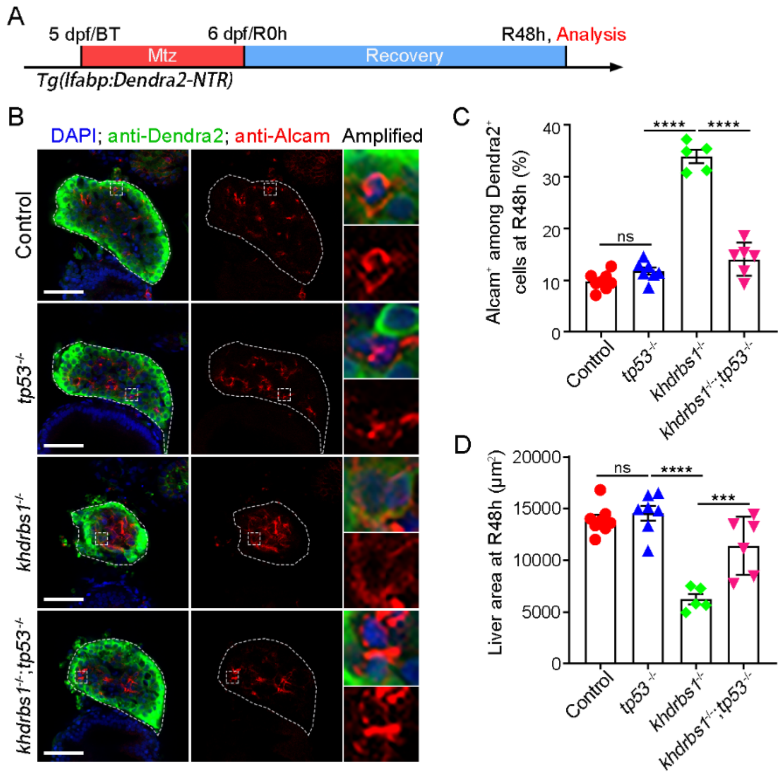

Fig. S12. *tp53* homozygous mutation in *khdrbs1$^{-/-}$* mutant partially rescues liver regeneration. (A) Scheme showing the stage of Mtz treatment and analysis at R48h. (B) Confocal images showing the expressions of Alcam and Dendra2 in livers after *tp53* homozygous mutation at R48h. (C, D) Quantification of the Alcam^{+} among Dendra2^{+} cells or the area of regenerating livers after *tp53* homozygous mutation at R48h. White-dashed circles point to livers. Scale bar: 50 μm. ns: not significant. Asterisks indicate statistical significance: ***$p < 0.001$, ****$p < 0.0001$. Error bars, ±SEM.

Table S1. Primer sequences used for probe synthesis in WISH and FISH

Available for download at
https://journals.biologists.com/dev/article-lookup/doi/10.1242/dev.204266#supplementary-data

Table S2. Primer sequences used for qPCR

Available for download at
https://journals.biologists.com/dev/article-lookup/doi/10.1242/dev.204266#supplementary-data

Development • Supplementary information
