# Supplementary material for: Khdrbs1 drives re-differentiation of bipotential progenitor cells by inhibiting p53 in zebrafish biliary-mediated liver regeneration
Source: Development. 2025 Feb 28;152(4):DEV204266. doi: 10.1242/dev.204266 (PMC11928054; doi:10.1242/dev.204266)
Supplement: Supplementary file 1 [file develop-152-204266_review_history.pdf]

Development

Khdrbs1 drives re-differentiation of bipotential progenitor cells by inhibiting p53 in zebrafish biliary-mediated liver regeneration

Kai Gang, Qi Chen, Junhui Sun, Tingwei Zhang, Pengcheng Cai, Rui Ni and Jianlong Ma

DOI: 10.1242/dev.204266

Editor: Ken Poss

Review timeline

Original submission:	21 July 2024
Editorial decision:	22 September 2024
First revision received:	3 December 2024
Editorial decision:	31 December 2024
Second revision received:	23 January 2025
Accepted:	26 January 2025

Original submission

First decision letter

MS ID#: dev.204266

MS TITLE: Khdrbs1 drives redifferentiation of bipotential progenitor cell by inhibiting p53 in zebrafish biliary-mediated liver regeneration

AUTHORS: Kai Gang, Qi Chen, Junhui Sun, Pengcheng Cai and Jianlong Ma

First, my apologies for the delay in this review and editorial process. I have now received all the referees' reports on the above manuscript, and have reached a decision. The referees' comments are appended below, or you can access them online: please go to:

As you will see, the referees express considerable interest in your work, but have some significant criticisms and recommend a substantial revision of your manuscript before we can consider publication. If you are able to revise the manuscript along the lines suggested, which may involve further experiments, I will be happy receive a revised version of the manuscript. Your revised paper will be re-reviewed by one or more of the original referees, and acceptance of your manuscript will depend on your addressing satisfactorily the reviewers' major concerns. Please also note that Development will normally permit only one round of major revision. If it would be helpful, you are welcome to contact us to discuss your revision in greater detail. Please send us a point-by-point response indicating your plans for addressing the referees' comments, and we will look over this and provide further guidance.

Please attend to all of the reviewers' comments and ensure that you clearly highlight all changes made in the revised manuscript. Please avoid using 'Tracked changes' in Word files as these are lost in PDF conversion. I should be grateful if you would also provide a point-by-point response detailing how you have dealt with the points raised by the reviewers in the 'Response to Reviewers' box. If you do not agree with any of their criticisms or suggestions please explain clearly why this is so.

Comments from the Reviewers:

© 2025. Published by The Company of Biologists under the terms of the Creative Commons Attribution License (https://creativecommons.org/licenses/by/4.0/).

Reviewer 1: This manuscript claims a novel role of Khdrbs1 in biliary-mediated liver regeneration in zebrafish, which is significant given its novelty. Both khdrbs1a and khdrbs1b were induced in BPPCs of regenerating zebrafish livers, and their deficiency impaired biliary-mediated liver regeneration, specifically, the proliferation and redifferentiation of BPPCs. In addition, the khdrbs1 mutant phenotypes were partially rescued in the tp53 heterozygous mutant background. These findings are well supported by the presented data in the manuscript. I have the following comments that should help improve the manuscript.

1. Rescue experiments were done only in the tp53 het background (Fig. 4). How about in the homo background? Better or no rescue?

2. For the rescue experiments, the proliferation of BPPCs was examined at R24h (Fig. 4B). How about at R8h?

3. In the regenerating livers of khdrbs1 mutants, which cell type(s) exhibited activated p53 signaling? Namely, which cell type expresses tp53, mdm2, or ccng?

4. Please speculate in the Discussion section how khdrbs1 deficiency activates p53 signaling in the regenerating livers.

Minor comments:
5. Provide the time window of the EdU treatment and its concentration.
6. Provide the working concentration of Dox.
7. Provide the working concentration of Cas9 protein and gRNA (p. 11).
8. Provide a reference showing ccng1 as a p53-associated gene.
9. The title of Fig. S2 is wrong.
10. Indicate what green means in Fig. 2C and Fig. 2D (upper). I assume anti-Dendra2 though.
11. There were no data showing activation of p53 signaling in khdrbs1 knockdown larvae (p. 5-6). "Activated p53 signaling" should be removed in the subtitle of the Result section in p. 5.
12. It was written in p. 8 "to evaluate p53 signaling according to clues from dCas9i system." However, such clues were not presented in the manuscript. Please explain the rationale to examine p53 signaling in khdrbs1 mutants.

Reviewer 2: In this manuscript Gang et al. used a zebrafish model of liver injury to study the role of khdrbs1 genes (very inconveniently named!) in liver regeneration. In particular, they focused on the process of dedifferentiation of biliary epithelial cells (BECs) into bipotential progenitor cells (BPPCs) and the redifferentiation of BPPCs into hepatocytes. In their previous work, they identified khdrbs1a and khdrbs1b regulation during liver regeneration. Here, they generated KO models of each gene to study their functions. However, these mutants showed normal liver regeneration, suggesting compensation.

While khdrbs1a and khdrbs1b mutants individually did not show liver regeneration defects, double knockout models (both dCas9i and genetic KO) showed impairment in regeneration. Expression of various markers showed that the redifferentiation step, not the dedifferentiation to BPPCs, was disrupted. The authors investigate p53 signaling and find that it is upregulated in the mutants and that ablating p53 signaling ameliorates the impairment in liver regeneration. The major strength of the paper is the use of several mutant fish lines to answer the key questions asked in the study. The major weakness results from a lack of explanation or clarity of rationale or experimental design. The text in the manuscript omits some key details which would improve reader comprehension and provide stronger support for the results.

Comments are included below.

Major Comments:

© 2025. Published by The Company of Biologists under the terms of the Creative Commons Attribution License (https://creativecommons.org/licenses/by/4.0/).

1. The authors need to briefly mention the specifics of liver injury. What is mtz? Why these developmental and post-injury time points? They only cite a previous study and describe it briefly in the methods but giving the method details and discussing the extent of liver injury, are important for readers to fully interpret the data and conclusions.
2. Figure S2B. Was there a compensatory increase in khdrbs1b in the khdrbs1a -/- mutants? Based on the isoform schematic, the frameshift mutation seems to occur in an isoform-specific manner.
3. Figure 2D. Cannot tell based on this image if complete colocalization is occurring or if the overlap is happening through the z-plane. Need higher magnification images.
4. The authors state, "Taken together, these data indicate that Khdrbs1 is indispensable for biliary-mediated liver regeneration." This is a strong statement. Did the KO fish regeneration fail completely causing lethality after 48h or was regeneration delayed?
5. The authors imply that the cells are held in a BPPC state, unable to dedifferentiate. Have the authors probed these cells from the single cell data to identify what stage(s) these cells are stuck in or any other factor(s) impeding their redifferentiation?
6. It is unclear from the main text why the authors decided to look at p53 signaling. Also, there are incorrect references to p53 when the data is not about p53 a few times in the manuscript. The section title on p.5 mentions p53, but the text does not discuss this at all. Fig S2 title mentions p53 yet I don't see how the figures show this. Fig S2B only shows whole-mount ISH of khdrbs1a.

Minor Comments:
1. Figure S1F. Arrows or circles to indicate differences in structure with a brief description in the figure legend would be helpful.
2. No figure 5. The text references Figure 5 panels A-E which are not included. Figure S5 is the correct figure according to the text.
3. The word "sibling" is used throughout the manuscript to refer to control animals. This is confusing, and they should change them to either "control" or a description of the exact genotype.

Reviewer 3: SUMMARY OF THE ADVANCE MADE IN THIS PAPER AND ITS POTENTIAL SIGNIFICANCE TO THE FIELD
Gang et al., generated khdrbs1a and khdrbs1b double mutants in zebrafish and demonstrated these two paralogues redundantly regulate liver regeneration in a biliary-mediated liver regeneration model. Mechanistically, they provide evidence showing that Khdrbs1a and Khdrbs1b function by promoting redifferentiation of BECs-derived bipotential progenitor cell (BPPCs) through repressing activation of p53 signaling during this process. The function of khdrbs1 genes in liver regeneration is a new discovery and the data are convincing; however, p53 signaling has been reported to play important roles in liver regeneration, and the mechanistic study is rather preliminary, e.g., there is no data on how Khdrbs1 repress the activation of p53 signaling.

SUGGESTIONS TO AUTHORS
Additional major comments:
1. The manuscript did not provide enough information on the essential concepts and research background, therefore it's difficult to evaluate the significance of this work.

(1) This manuscript mainly concerns "redifferentiation of bipotential progenitor cell" in "biliary-mediated liver regeneration". However, there is little introduction on these two concepts in the section of INTRODUCTION, and the background introduction is instead largely focused on ductular reaction (DR) and BEC transdifferentiation. Does it have anything to do with "redifferentiation of bipotential progenitor cell" and "biliary-mediated liver regeneration"?

(2) BPPCs is an important cell type used in this manuscript, the authors should give the full name and enough background introduction, such as its origin, molecular and cellular features and function (especially in liver regeneration), in its first appearance in the main text.

(3) What does "biliary-mediated liver regeneration" mean? When and how is the liver injury achieved? Why the regeneration is "biliary-mediated"? How long it will take for this liver regeneration to be complete? The authors should give a clear introduction to this regeneration

© 2025. Published by The Company of Biologists under the terms of the Creative Commons Attribution License (https://creativecommons.org/licenses/by/4.0/).

model, compare it with other liver regeneration models, and explain why this model was chosen for this study. Particularly, what is the relationship between "biliary-mediated liver regeneration" and BPPCs?

(4) The authors should provide enough information on khdrbs1 gene in the section of INTRODUCTION. For example, what is the molecular feature of khdrbs1 gene product, e.g., type of protein (an enzyme? a transcription factor? a receptor?), molecular function (substrate? target gene? ligand?), etc.

2. The authors "observed that both khdrbs1 paralogues were significantly upregulated in BPPCs at regeneration 0 hour (R0h)": This means upregulation of khdrbs1 happened before R0h, when is the earliest time point of khdrbs1 upregulation after MTZ treatment? When BPPCs emerge during the liver regeneration?

3. Fig. 1B: The cells other than those in the BPPC population also showed obvious increase in khdrbs1a expression in the R0h sample, what are the identity of these cells? How is the expression of lfabp, tp1 and Dendra2 in these single-cell RNA sequencing data?

4. Fig. 1B and 1C: The authors "observed that both khdrbs1 paralogues were significantly upregulated in BPPCs at regeneration 0 hour (R0h), particularly khdrbs1a, through our previous single-cell RNA sequencing data (Cai et al., 2021) (Fig. 1A, B)". "WISH results showed that khdrbs1a was apparently upregulated in the liver region at R8h and R24h (Fig. 1C)": These two results are somewhat inconsistent? Why the WISH result of khdrbs1a at R0h did not show higher expression than the control as the single-cell RNA sequencing data? qRT-PCR should be a more sensitive and quantitative measurement on this issue.

5. Fig. 1D: "fluorescence in situ hybridization (FISH) coupled with antibody staining assays revealed that both khdrbs1a and khdrbs1b were mainly expressed in BPPCs co-expressed GFP and Dendra2 at R8h and R24h (Fig. 1D)": How is khdrbs1a expression at R0h evaluated using this method?

6. The authors claim "both khdrbs1a and khdrbs1b were mainly expressed in BPPCs co-expressed GFP and Dendra2 at R8h and R24h (Fig. 1D)." It seems the authors are using GFP (=red fluorescent signal) and Dendra2 as the markers for BPPCs? However, there are many green (not GFP) cells (=expressing only khdrbs1a without GFP or Dendra2?) at R8h and R24h for the panel of FISH-khdrbs1a, these cells are quite abundant but should not belong to BPPCs, so it's not precise to claim that khdrbs1a is mainly expressed in BPPCs? What are the identity of these green cells?

7. Fig S2B: "WISH images showing the expression patterns of khdrbs1a and tp53 in control and dCas9i group at R24h." However, no tp53 expression results were given in this figure.

8. khdrbs1a and khdrbs1b are expressed in liver at 3.5 dpf (Fig. S1) but not at 6 dpf (Fig. 1C and 1D), what are their expression dynamics during liver development, i.e., When they start their liver expression and when they stop liver expression?

9. Fig. 2D, lower panel: Does anti-GFP indicate Tg(Tp1: GFP) signal (labeling intrahepatic BECs)? Why there is no GFP signal (= no BECs?) in the control embryo?

10. "The regenerating livers of dCas9iS subgroup co-expressed GFP and Dendra2 at R48h, demonstrating that this dCas9i system is BECs-specific for biliary-mediated liver regeneration (Fig. 2D, bottom panel)": This sentence is difficult to understand, why co-expression of GFP and Dendra2 in the regenerating livers could demonstrate the dCas9i system is BECs-specific?

11. The subtitle "Specific knockdown of khdrbs1 in BECs results in impaired biliary-mediated liver regeneration and activated p53 signaling": However, no experimental data concerning p53 signaling were mentioned in this section.

© 2025. Published by The Company of Biologists under the terms of the Creative Commons Attribution License (https://creativecommons.org/licenses/by/4.0/).

12. "khdrbs1 mutants exhibited a significant upregulation of tp53, mdm2, and ccng1 across broad anatomical regions including optic tectum, pharyngeal pouches, and gut, but not in the liver at 5 dpf (Fig. S5A)": It's interesting that the expression of tp53, mdm2, and ccng1 is activated in some specific embryonic regions but not liver in the khdrbs1 mutants, does it mean khdrbs1a and khdrbs1b are not expressed in liver at 5 dpf? Will tp53, mdm2, and ccng1 expression be induced in khdrbs1 mutant liver at 3.5 dpf, when khdrbs1a and khdrbs1b are present in WT liver at this stage (and might be required to repress p53 in liver at this stage)?

13. Fig. S5C vs Fig. 4A: Why tp53 was not upregulated as shown by q-PCR (Fig. S5C) but increased in the liver as shown by WISH (Fig. 4A)?

14. "Further investigations of WISH analyses indicated that tp53 and mdm2 were significantly upregulated in the regenerating liver of khdrbs1 mutants at R8h (Fig. S5B)": p53 signaling upregulation seems quite early in khdrbs1 mutants after liver injury. How early is it activated? Khdrbs1 seem to be responsible to repress p53 activation during the liver regeneration. Is there any correlation on the timing between khdrbs1 upregulation in WT embryos and p53 signaling activation in khdrbs1 mutants?

15. Fig. 4B-4F: The authors should provide the result of tp53+/- as a control.

16. "the proliferation rate of BPPCs was partially rescued by the heterozygous tp53 (tp53+/-) in khdrbs1 mutants (Fig. 4B)": However, this figure showed the ratio of EdU+ cells among Dendra2+ cells (representing hepatocytes), which cannot demonstrate proliferation of BPPCs.

17. Heterozygous tp53 (tp53+/-) partially rescued liver regeneration defects of the khdrbs1 mutants (Fig. 4B-4F), could p53 homozygous mutant give better rescue effects?

Minor comments:
1. Fig. 1A, 1D, 2B, 3A, 3D, 4B, S2A, S3A, S4A: What does "BT" mean?

2. Fig. 1B: The color of the scale bar (from 0.0 to 9.0) starts with orange (0.0) and ends with red (9.0), does it represent relative level of gene expression and 0.0 means no expression? If yes, why there are grey-colored cells in the UMAP?

3. Fig. 1B: "Lgo2 Exp" seems should be "Log2 Exp"?

4. Fig S2A: (1) Indicate the transgenic fish used? (2) Right panel: "Lver area at R48h" should be "Liver area at R48h"?

5. "The expressions of tp53, mdm2 and ccng1 were also highly increased in the regenerating livers of khdrbs1 mutants at R24h (Fig. 5A)": Fig. 5A should be Fig. 4A?

6. "the proportion of Alcam+ cells among Dendra2+ cells was diminished by tp53 mutation in khdrbs1 mutants (Fig. 5C and D), indicating that the redifferentiation of BPPCs was rescued. Moreover, tp53 mutation also partially rescued the regenerating liver size of khdrbs1 mutants at R48h (Fig. 5E), as well as the expression of hepatocyte markers bhmt and hnf4a (Fig. 5F)": Fig. 5C through 5F should be Fig. 4 C through 4F?

7. English grammar needs improvements, e.g., "khdrbs1a was decreased expression in regenerating livers of dCas9i group".

8. The citation of some reference is incomplete, e.g., the one of Fu, K. et al.

© 2025. Published by The Company of Biologists under the terms of the Creative Commons Attribution License (https://creativecommons.org/licenses/by/4.0/).

First revision

Author response to reviewers' comments

Point by point response to the reviewers

Response to Reviewer #1

Reviewer 1: This manuscript claims a novel role of Khdrbs1 in biliary-mediated liver regeneration in zebrafish, which is significant given its novelty. Both khdrbs1a and khdrbs1b were induced in BPPCs of regenerating zebrafish livers, and their deficiency impaired biliary-mediated liver regeneration, specifically, the proliferation and redifferentiation of BPPCs. In addition, the khdrbs1 mutant phenotypes were partially rescued in the tp53 heterozygous mutant background. These findings are well supported by the presented data in the manuscript. I have the following comments that should help improve the manuscript.

Response: We would like to express our sincere gratitude for your constructive and insightful comments. Your suggestions have greatly contributed to improving the quality of our manuscript. We have carefully considered your comments and revised the manuscript accordingly. We hope that all the corrections and additional experimental results adequately address the concerns you have raised.

1. Rescue experiments were done only in the tp53 het background (Fig. 4). How about in the homo background? Better or no rescue?

Response: Thank you for your thoughtful comments. We evaluated the redifferentiation and liver size of the *khdrbs1*$^{-/-}$;*tp53*$^{-/-}$ mutants. Consistent with the rescue effects observed in the *tp53* heterozygous mutants (Fig. 4D-4F in the revised Figure 4), the *tp53* homozygous mutation also partially rescued the defective phenotypes of the *khdrbs1*$^{-/-}$ mutant, resulting in phenotypes comparable to those observed in the *tp53* heterozygous mutation (Fig. S10A- S10D in the revised Supplementary Information).

2. For the rescue experiments, the proliferation of BPPCs was examined at R24h (Fig. 4B). How about at R8h?

Response: Thank you for your constructive question. EdU assay was performed to validate the proliferation of BPPCs in the *tp53*$^{+/-}$;*khdrbs1*$^{-/-}$ mutant at R8h. In contrast to the rescue effects observed at R24h (Fig. 4C in the revised Figure 4), the results of EdU labeling and quantification showed that *tp53* mutation did not significantly rescue the proliferation of BPPCs in *khdrbs1*$^{-/-}$ mutants at R8h (Fig. S9D in the revised Supplementary Information). However, a few of the *tp53*$^{+/-}$;*khdrbs1*$^{-/-}$ mutants exhibited enhanced BPPC proliferation compared to the *khdrbs1*$^{-/-}$ mutants (Fig. S9D in the revised Supplementary Information), suggesting that the *tp53* mutation may have a subtle but not obvious effect at R8h.

3. In the regenerating livers of khdrbs1 mutants, which cell type(s) exhibited activated p53 signaling? Namely, which cell type expresses tp53, mdm2, or ccng?

Response: Thank you for your helpful comments. We detected the expression of *khdrbs1a* and *khdrbs1b* at R0h after severe liver injury. FISH of *khdrbs1* assays showed that *khdrbs1* was primarily expressed in Anax4^{+} BPPCs, while *khdrbs1a* expression also increased in other cells (e.g., macrophages and hepatic stellate cells) at R0h (Fig. S2C-S2D and Fig. S3A-S3C in the revised Supplementary Information). Moreover, the p53 antibody and *tp53* RNA probe were used to assess p53 expression in *khdrbs1*$^{-/-}$ mutants at R8h. Immunofluorescence and FISH assays showed that

© 2025. Published by The Company of Biologists under the terms of the Creative Commons Attribution License (https://creativecommons.org/licenses/by/4.0/).

most of the Anax4^{+} BPPCs were labeled by p53 in *khdrbs1*$^{-/-}$ mutants at R8h, whereas some Anax4^{-} cells were also labeled by p53 (Fig. 4B in the revised Figure 4 and Fig. S9C in the revised Supplementary Information). These results suggest that p53 signaling responds to the loss of *khdrbs1* from BPPCs and other non-hepatic cells during biliary-mediated liver regeneration.

4. Please speculate in the Discussion section how khdrbs1 deficiency activates p53 signaling in the regenerating livers.

Response: We appreciate the reviewer for this insightful suggestion. We have hypothesizedtheinteractionbetweenKhdrbs1andp53andhaveincludeditin the "RESULTS AND DISCUSSION"sectionoftherevisedmanuscript.Given theaccumulationofp53inthenucleusofBPPCsofthe *khdrbs1*$^{-/-}$ mutant(Fig. 4BintherevisedFigure4),wespeculatethatKhdrbs1mayinfluencep53 stability.

Minor comments:

5. Provide the time window of the EdU treatment and its concentration.

Response: Thank you for pointing this out. We apologize for the oversight and have now included a detailed description of EdU treatment, including its time window and working concentration, in the "MATERIALS AND METHODS" section of the revised manuscript.

6. Provide the working concentration of Dox.

Response: We thank the reviewer for pointing out this oversight. We apologize for the oversight and have now included the working concentration of Dox in the section of "MATERIALS AND METHODS" in the revised manuscript.

7. Provide the working concentration of Cas9 protein and gRNA (p. 11).

Response: Thank you for pointing out this oversight. We apologize for the oversight and have added the working concentration of Cas9 protein and gRNA in the "MATERIALS AND METHODS" section.

8. Provide a reference showing ccng1 as a p53-associated gene.

Response: Thank you for raising this important point. *ccng1* is recognized as one of the p53 target genes (Chen et al., 2005; Okamoto and Beach, 1994). The relevant references have been cited in the revised manuscript (line 236).

9. The title of Fig. S2 is wrong.

Response:Thankyouforpointingthismistake.Weapologizefortheoversight andhavecorrectedthetitlein Fig.S5intherevisedSupplementaryInformation.

10. Indicate what green means in Fig. 2C and Fig. 2D (upper). I assume anti- Dendra2 though.

Response: Thank you for your kind reminder. We apologize for any confusion caused by these images. These green signals represent the fluorescence of livers in live larvae rather than the immunofluorescence via anti-Dendra2. We have added the detailed information of the transgenic fish (Fig. 2B in the revised Figure 2). The epifluorescence images are also taken from live larvae and mainly show the body shape (Fig. 2C in the revised Figure 2).

11. There were no data showing activation of p53 signaling in khdrbs1 knockdown larvae (p. 5-6). "Activated p53 signaling" should be removed in the subtitle of the Result section in p. 5.

Response: We thank the reviewer for bringing this to our attention and apologize for our

© 2025. Published by The Company of Biologists under the terms of the Creative Commons Attribution License (https://creativecommons.org/licenses/by/4.0/).

oversight. The data of activation of p53 signaling in the *khdrbs1* knockdown larvae have been added in the revised Supplementary Information (Fig. S8D and S9A-S9B in the revised Supplementary Information). We also have corrected and rephrased these data in the revised manuscript.

12. It was written in p. 8 "to evaluate p53 signaling according to clues from dCas9i system." However, such clues were not presented in the manuscript. Please explain the rationale to examine p53 signaling in khdrbs1 mutants.

Response: We appreciate the reviewer for highlighting this important point. In the revised manuscript, we have corrected and further clarified this point. Based on the significantly defective phenotypes of liver regeneration observed after loss of *khdrbs1*, characterized by normal dedifferentiation and impaired redifferentiation, and our previous findings about p53 in liver regeneration (He et al., 2022; Song et al., 2023), we decided to evaluate the p53 signaling. Our results showed that p53 signaling was activated in our liver regeneration model after loss of *khdrbs1* (lines 233-236).

Response to Reviewer #2

Reviewer 2: In this manuscript Gang et al. used a zebrafish model of liver injury to study the role of khdrbs1 genes (very inconveniently named!) in liver regeneration. In particular, they focused on the process of dedifferentiation of biliary epithelial cells (BECs) into bipotential progenitor cells (BPPCs) and the redifferentiation of BPPCs into hepatocytes. In their previous work, they identified khdrbs1a and khdrbs1b regulation during liver regeneration. Here, they generated KO models of each gene to study their functions. However, these mutants showed normal liver regeneration, suggesting compensation.

While khdrbs1a and khdrbs1b mutants individually did not show liver regeneration defects, double knockout models (both dCas9i and genetic KO) showed impairment in regeneration. Expression of various markers showed that the redifferentiation step, not the dedifferentiation to BPPCs, was disrupted. The authors investigate p53 signaling and find that it is upregulated in the mutants and that ablating p53 signaling ameliorates the impairment in liver regeneration. The major strength of the paper is the use of several mutant fish lines to answer the key questions asked in the study. The major weakness results from a lack of explanation or clarity of rationale or experimental design. The text in the manuscript omits some key details which would improve reader comprehension and provide stronger support for the results.

Response: We sincerely appreciate the reviewer's thoughtful feedback and insightful suggestions, which have greatly enhanced the clarity and depth of our study. In response, we have made substantial revisions to the manuscript, including additional experiments and analyses, to further strengthen the support for our main conclusions. Please find below the detailed point-by-point responses, along with the revised manuscript and supporting information (blue text).

Comments are included below.

Major Comments:

1. The authors need to briefly mention the specifics of liver injury. What is mtz? Why these developmental and post-injury time points? They only cite a previous study and describe it briefly in the methods but giving the method details and discussing the extent of liver injury, are important for readers to fully interpret the data and conclusions.

Response: Thank you for your constructive comments. Following your suggestions, we have incorporated details about our liver injury model, including the role of Mtz and the rationale for selecting specific developmental and post-injury time points, in the revised manuscript (lines 63-76, 106-110).

2. Figure S2B. Was there a compensatory increase in khdrbs1b in the khdrbs1a -/- mutants? Based on

© 2025. Published by The Company of Biologists under the terms of the Creative Commons Attribution License (https://creativecommons.org/licenses/by/4.0/).

the isoform schematic, the frameshift mutation seems to occur in an isoform-specific manner.

Response: Thank you for your helpful comment. We evaluated the expression of *khdrbs1b* in *khdrbs1a*$^{-/-}$ mutants at R24h. WISH results showed increased *khdrbs1b* in the brain and regenerating liver of *khdrbs1a*$^{-/-}$ mutants, suggesting genetic compensation of *khdrbs1a* and *khdrbs1b* during liver regeneration (Fig. S5C in the revised Supplementary Information).

3. Figure 2D. Cannot tell based on this image if complete colocalization is occurring or if the overlap is happening through the z-plane. Need higher magnification images.

Response: Thank you for your kind reminder. We agree with the comment and have corrected this data using 2-dimentional images and higher magnification images (Fig. 2C in the revised Figure 2). These updated data indicate that the GFP signal induced by Dox treatment overlaps with Dendra2 at R48h.

4. The authors state, "Taken together, these data indicate that Khdrbs1 is indispensable for biliary-mediated liver regeneration." This is a strong statement. Did the KO fish regeneration fail completely causing lethality after 48h or was regeneration delayed?

Response: Thank you for your constructive questions. We validated the control and the *khdrbs1*$^{-/-}$ mutant at the late stage of liver regeneration (R48h-R96h).
The regenerating liver of the *khdrbs1*$^{-/-}$ mutant did not recover at R48h and R72h (Fig. S7H in the revised Supplementary Information). Survival rates of the control and *khdrbs1*$^{-/-}$ mutant were quantified and showed that the *khdrbs1*$^{-/-}$ mutant had higher lethality at R72h and R96h compared to the control (Fig. S7H in the revised Supplementary Information). These data indicate that the *khdrbs1*$^{-/-}$ mutant fails to regenerate the liver rather than delaying it, reinforcing the indispensable role of Khdrbs1 in liver regeneration.

5. The authors imply that the cells are held in a BPPC state, unable to dedifferentiate. Have the authors probed these cells from the single cell data to identify what stage(s) these cells are stuck in or any other factor(s) impeding their redifferentiation?

Response: Thank you for your helpful comments. Our results suggest that BECs normally express the hepatoblast markers during liver regeneration after loss of *khdrbs1*, indicating that the deficiency of khdrbs1 does not impact the dedifferentiation of BECs after liver injury (Fig. S6B-S6C in the revised Supplementary Information).
We apologize for not having identified the precise characteristics of BPPCs after loss of *khdrbs1* by single-cell RNA sequencing to determine the exact stages in this paper. However, using RNA probes of liver regeneration associated genes, WISH analysis showed that these genes were scarcely expressed in BPPCs deficient in *khdrbs1* during liver regeneration, suggesting impaired the redifferentiation (Fig. S6D-S6E in the revised Supplementary Information).

6. It is unclear from the main text why the authors decided to look at p53 signaling. Also, there are incorrect references to p53 when the data is not about p53 a few times in the manuscript. The section title on p.5 mentions p53, but the text does not discuss this at all. Fig S2 title mentions p53 yet I don't see how the figures show this. Fig S2B only shows whole-mount ISH of khdrbs1a.

Response: Thank you for pointing this issue out. 1) Based on the significantly defective phenotypes of liver regeneration observed after loss of *khdrbs1*, characterized by normal dedifferentiation and impaired redifferentiation, and our previous findings about p53 in liver regeneration (He et al., 2022; Song et al., 2023), we decided to evaluate the p53 signaling. Our results showed that p53 signaling was activated in our liver regeneration model after loss of *khdrbs1* (lines 233-236). 2) We have carefully proofread the manuscript and corrected the references to p53, ensuring they are

© 2025. Published by The Company of Biologists under the terms of the Creative Commons Attribution License (https://creativecommons.org/licenses/by/4.0/).

accurately linked to the data presented (lines 266-273). 3) We have addressed the discrepancies and added the correct data (Fig. S8D in the revised Supplementary Information), as well as updated the relevant discussion in the revised manuscript.

Minor Comments:

1. Figure S1F. Arrows or circles to indicate differences in structure with a brief description in the figure legend would be helpful.

Response: Thank you for your kind reminder. Red arrows and white dashed circles have been added to highlight the liver. We have also provided a brief description of these differences in the figure legend in the revised manuscript and Supplementary Information.

2. No figure 5. The text references Figure 5 panels A-E which are not included. Figure S5 is the correct figure according to the text.

Response: Thank you for carefully reading our manuscript and pointing this mistake. The figure referenced as Figure 5 in the text is actually Figure 4. We apologize for this oversight and have corrected the errors in the revised manuscript.

3. The word "sibling" is used throughout the manuscript to refer to control animals. This is confusing, and they should change them to either "control" or a description of the exact genotype.

Response: We thank the reviewer for this valuable suggestion. To avoid confusion, we have replaced the term "sibling" with "control" in the revised manuscript, figures, and Supplementary Information accordingly.

Response to Reviewer #3

Reviewer 3: SUMMARY OF THE ADVANCE MADE IN THIS PAPER AND ITS POTENTIAL SIGNIFICANCE TO THE FIELD
Gang et al., generated khdrbs1a and khdrbs1b double mutants in zebrafish and demonstrated these two paralogues redundantly regulate liver regeneration in a biliary-mediated liver regeneration model. Mechanistically, they provide evidence showing that Khdrbs1a and Khdrbs1b function by promoting redifferentiation of BECs-derived bipotential progenitor cell (BPPCs) through repressing activation of p53 signaling during this process. The function of khdrbs1 genes in liver regeneration is a new discovery and the data are convincing; however, p53 signaling has been reported to play important roles in liver regeneration, and the mechanistic study is rather preliminary, e.g., there is no data on how Khdrbs1 repress the activation of p53 signaling.

Response: Thank you for your thoughtful comments and for highlighting the significance of our study. We appreciate your recognition of the novel role of *khdrbs1* gene in liver regeneration and acknowledge that the mechanistic data on Khdrbs1 regulation of p53 signaling are preliminary. We agree that further exploration is needed to fully elucidate the underlying mechanisms. Weare grateful for your valuable feedback, which has helped us improve the manuscript.

SUGGESTIONS TO AUTHORS
Additional major comments:
1. The manuscript did not provide enough information on the essential concepts and research background, therefore it's difficult to evaluate the significance of this work.
(1) This manuscript mainly concerns "redifferentiation of bipotential progenitor cell" in "biliary-mediated liver regeneration". However, there is little introduction on these two concepts in the section of INTRODUCTION, and the background introduction is instead largely focused on ductular reaction (DR) and BEC transdifferentiation. Does it have anything to do with "redifferentiation of bipotential progenitor cell" and "biliary-mediated liver regeneration"?

© 2025. Published by The Company of Biologists under the terms of the Creative Commons Attribution License (https://creativecommons.org/licenses/by/4.0/).

Response:Wethankthereviewerfortheseconstructivecomments.Wehave revised the " INTRODUCTION" section to better clarify the concepts of "redifferentiation of bipotential progenitor cells" and "biliary-mediated liver regeneration."Wehavealsoadjustedthedescriptionsofductularreaction(DR) and BECtransdifferentiationtoensureaclearerdistinctionandrelevancetothe main focusofthemanuscript(lines50-56,63-76).DRoftenaccompaniesliver diseasesin whichtheBECsproliferateandbileductsareincreased.Whenthe hepatocytesare inhibited not enough to compensateforliverinjury,BECsfrom DRproliferateandtransdifferentiateintohepatocytes.

(2) BPPCs is an important cell type used in this manuscript, the authors should give the full name and enough background introduction, such as its origin, molecular and cellular features and function (especially in liver regeneration), in its first appearance in the main text.

Response: Thank you for your kind reminder. We have added a detailed description of BPPCs, including its full name, origin, molecular and cellular features, and role in liver regeneration, in the "INTRODUCTION"sectionofthe revisedmanuscript(lines69-76).

(3) What does "biliary-mediated liver regeneration" mean? When and how is the liver injury achieved? Why the regeneration is "biliary-mediated"? How long it will take for this liver regeneration to be complete? The authors should give a clear introduction to this regeneration model, compare it with other liver regeneration models, and explain why this model was chosen for this study. Particularly, what is the relationship between "biliary-mediated liver regeneration" and BPPCs?

Response: We thank the reviewer for these helpful comments. We have revised and expanded the description of our liver regeneration model in the revised manuscript (lines 63-76, 106-110) and Fig.S1 in the Supplementary Information.
1) Biliary-mediated liver regeneration refers to liver regeneration driven by biliary epithelial cells (BECs), which are the main origin of nascent hepatocytes and contribute to liver regeneration following severe liver injury. 2) Liver injury is induced using the Nitroreductase (NTR)/metronidazole (Mtz) system, which ablates hepatocytes in zebrafish at 5 dpf, creating a model of severe liver injury.
3) In this model, hepatocytes are almost completely ablated, and liver regeneration primarily occurs through transdifferentiation of BECs into nascent hepatocytes. This process is referred to as biliary-mediated liver regeneration.
4) According to previous reports, the liver requires at least 72 hours to regenerate to its pre-injury level after NTR/Mtz-induced injury. 5) Compared with other liver regeneration models, in which the liver mainly regenerates through hepatocyte proliferation, the most important advantages of our model are almost eliminating the regeneration from hepatocytes. This allows for a more focused investigation of alternative sources of nascent hepatocytes, particularly BECs. According to our previous single-cell RNA sequencing data, we observed upregulation of *khdrbs1* in BPPCs. We aim to further explore the role of Khdrbs1 in the liver progenitor cells during liver regeneration, therefore we used the model of biliary-mediated liver regeneration in this paper. 6) After severe liver injury in our model, BECs contribute to liver regeneration by dedifferentiating into bipotential progenitor cells (BPPCs), which then redifferentiate into hepatocytes.

(4) The authors should provide enough information on khdrbs1 gene in the section of INTRODUCTION. For example, what is the molecular feature of khdrbs1 gene product, e.g., type of protein (an enzyme? a transcription factor? a receptor?), molecular function (substrate? target gene? ligand?), etc.

Response:Wethankthereviewerforpointingthisissueout.Wehaveadded detailed information on the Khdrbs1 gene, including its molecular features, protein type, and molecular function, in the "INTRODUCTION"section(lines 83-94).

2. The authors "observed that both khdrbs1 paralogues were significantly upregulated in BPPCs at

© 2025. Published by The Company of Biologists under the terms of the Creative Commons Attribution License (https://creativecommons.org/licenses/by/4.0/).

regeneration 0 hour (R0h)": This means upregulation of khdrbs1 happened before R0h, when is the earliest time point of khdrbs1 upregulation after MTZ treatment? When BPPCs emerge during the liver regeneration? (R0h?)

Response: Thank you for your constructive comments. 1) We detected the expression of *khdrbs1a* and *khdrbs1b* via FISH at multiple time points: 5 dpf (before Mtz treatment), Mtz-16h (late stage of Mtz treatment), and R0h (stopping Mtz treatment). The FISH analysis revealed that the expression of *khdrbs1a* was significantly increased in Anxa4^{+} BECs at both Mtz-16h and R0h, whereas *khdrbs1b* was primarily upregulated in Anxa4^{+} BECs at R0h (Fig. S2C- intherevisedSupplementaryInformation).We found that the *khdrbs1* has significant expression in partial Anxa4^{+} BECs at Mtz-16h after Mtz treatment. Theseresultsshowedthattheincreaseof *khdrbs1* isinresponsetosevereliver injury. 2) BPPCs are derived from BECs and acquire the characteristics of hepatoblasts (*sox9b*, *foxa3*, and *hhex*) and then transdifferentiate to hepatocytes and BECs (He et al., 2014) after severe liver injury. BPPCs emerged during severe liver injurywhenhepatocyteswereinsufficienttorepair theliver(Choietal.,2014),rather thanspecificallyatR0h.TovalidateBECsfor liverregeneration,wechoosethestageofR0h,whichmeansthat almostallof hepatocytesareablatedandminimizestheimpactonliverregenerationby hepatocytes.

3. Fig. 1B: The cells other than those in the BPPC population also showed obvious increase in khdrbs1a expression in the R0h sample, what are the identity of these cells? How is the expression of lfabp, tp1 and Dendra2 in these single-cell RNA sequencing data?

Response: We appreciate the reviewer for these insightful questions. 1) In Fig.1B of the revised Figure 1, the cells other than those in the BPPC population include hepatic stellate cells (HSC), vascular epithelial cells (VEC), and immune cells (IC), excluding the BEC, hepatocyte and BPPC populations (Cai et al., 2021). These annotations are now indicated in the revised manuscript. Based on single-cell RNA sequencing data, the expression of *khdrbs1a* is indeed increased in HSC, IC and VEC.

2)The single-cellRNAsequencingdataof*lfabp*and*Dendra2*expressionare shown in the following figure (FigureA).Theexpressionpatternsof*lfabp*and *Dendra2*areconsistent,withbothbeinghighlyexpressedin the hepatocyte population at 6 dpf. Moreover, a slight increase in *lfabp* and *Dendra2* expression inresponsetoNotchsignalingandisusedtolabelthebileductsinzebrafish liver (Ninov et al., 2012). In addition, the single-cell RNA sequencing data of *her9* (hairy-related9),oneoftheNotchtargetgenes,andBECsmarker*anxa4* arealso providedinthefollowingfigure(FigureB).Thesedatashowthat*her9* and*anxa4*are significantly expressed inBECsorBPPCs.

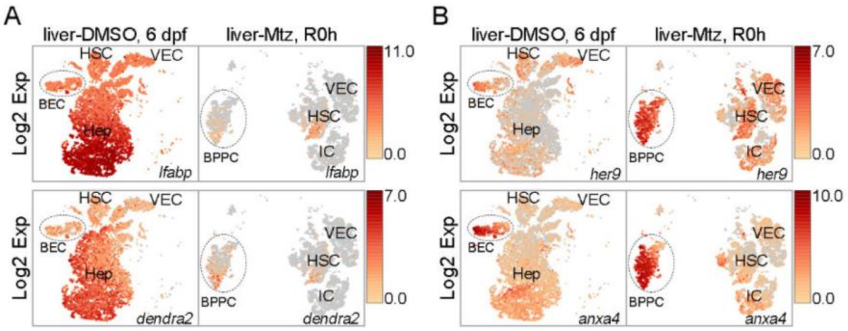

Uniform manifold approximation and projection (UMAP) showing the expressionof*lfabp*,*Dendra2*, *her9*and*anxa4*(overlayedred)inliversat6dpf andR0h,respectively.

4. Fig. 1B and 1C: The authors "observed that both khdrbs1 paralogues were significantly upregulated in BPPCs at regeneration 0 hours (R0h), particularly khdrbs1a, through our previous

© 2025. Published by The Company of Biologists under the terms of the Creative Commons Attribution License (https://creativecommons.org/licenses/by/4.0/).

single-cell RNA sequencing data (Cai et al., 2021) (Fig. 1A, B)". "WISH results showed that khdrbs1a was apparently upregulated in the liver region at R8h and R24h (Fig. 1C)": These two results are somewhat inconsistent? Why the WISH result of khdrbs1a at R0h did not show higher expression than the control as the single-cell RNA sequencing data? qRT-PCR should be a more sensitive and quantitative measurement on this issue.

Response: We thank the reviewer for these thoughtful comments and valuable suggestions. This inconsistency may be caused by the different sensitivity between single-cell RNA sequencing and WISH, as WISH tends to be less results showed that the significant expression of *khdrbs1a* at R0h and comparable expression of *khdrbs1b* at R0h, compared to the expressions at 6 dpf (Fig. S2A-S2B in the revised Supplementary Information). These data are consistent with the results from single-cell RNA sequencing. Moreover, *khdrbs1a* and *khdrbs1b* were significantly expressed at R8h (Fig. S2B in the revised Supplementary Information).

5. Fig. 1D: "fluorescence in situ hybridization (FISH) coupled with antibody staining assays revealed that both khdrbs1a and khdrbs1b were mainly expressed in BPPCs co-expressed GFP and Dendra2 at R8h and R24h (Fig. 1D)": How is khdrbs1a expression at R0h evaluated using this method?

Response: Thank you for your helpful question. Using anti-Anxa4 to label BECs, we performed FISH and antibody staining to detect the expression of *khdrbs1a* and *khdrbs1b* at 6dpf, Mtz-16h and R0h, respectively. These results demonstrate that the expression of *khdrbs1a* and *khdrbs1b* are upregulated in Anxa4^{+} BECs after severe liver injury (Fig. S2C-S2D in the revised Supplementary Information). However, the Dendra2 signal cannot be detected in BECs at R0h (Fig. S2C-S2D in the revised Supplementary Information). Notably, R0h is a time when almost all of hepatocytes are ablated and BECs have acquired the characteristics of hepatoblasts (He et al., 2014) rather than those of mature hepatocytes.

6. The authors claim "both khdrbs1a and khdrbs1b were mainly expressed in BPPCs co-expressed GFP and Dendra2 at R8h and R24h (Fig. 1D)." It seems the authors are using GFP (=red fluorescent signal) and Dendra2 as the markers for BPPCs? However, there are many green (not GFP) cells (=expressing only khdrbs1a without GFP or Dendra2?) at R8h and R24h for the BPPCs? What is the identity of these green cells?

Response: We appreciate the reviewer for these insightful comments. 1) Indeed, the signals of GFP (red) and Dendra2 (grey) are used to label BPPCs (Fig. 1D in the revised figure). 2) Due to the significant expression of *khdrs1a* at R0h, we quantified the percentage of Anxa4^{+} BECs, lcp1^{+} macrophages, and hand2^{+} hepatic stellate cells among FISH-*khdrs1a*$^{+}$ cells at R0h, respectively. Anxa4^{+} and FISH-*khdrs1a*$^{+}$ co-expressing cells account for 65%, and lcp1^{+} macrophages account for approximately 27% among FISH-*khdrs1a*$^{+}$ cells (Fig. S3A-S3C in the revised Supplementary Information). These data indicate that *khdrbs1a* is mainly expressed in BECs after liver injury.
Moreover, we quantified the percentage of GFP^{+} Dendra2^{+} FISH-*khdrs1a*$^{+}$ cells, GFP^{-} Dendra2^{+} FISH-*khdrs1a*$^{+}$ cells, and GFP^{-} Dendra2^{-} FISH-*khdrs1a*$^{+}$ cells among FISH-*khdrs1a*$^{+}$ cells at R8h and R24h, respectively. GFP^{+} Dendra2^{+} FISH-*khdrbs1a*$^{+}$ cells account for 78% at R8h and 83% at R24h, respectively, suggesting that *khdrbs1a* is mainly expressed in GFP and Dendra2 co- expressed BPPCs (Fig. S3D in the revised Supplementary Information).
Some cells are GFP^{-} Dendra2^{+} FISH-*khdrs1a*$^{+}$ at R8h and R24h, which may imply the incomplete labelling of BECs by the transgenic line *Tg(Tp1:GFP)*. Furthermore, according to different FISH-*khdrs1a*$^{+}$ cells at R0h, GFP^{-} Dendra2^{-} FISH-*khdrs1a*$^{+}$ cells may include both macrophages and hepatic stellate cells at R8h and R24h.

7. Fig S2B: "WISH images showing the expression patterns of khdrbs1a and tp53 in control and

© 2025. Published by The Company of Biologists under the terms of the Creative Commons Attribution License (https://creativecommons.org/licenses/by/4.0/).

dCas9i group at R24h." However, no tp53 expression results were given in this figure.

Response: We thank the reviewer for pointing out this oversight. We have corrected this error and descripteditintherevisedmanuscript(Fig.S8Dinthe revisedSupplementaryInformation).

8. khdrbs1a and khdrbs1b are expressed in liver at 3.5 dpf (Fig. S1) but not at 6 dpf (Fig. 1C and 1D), what are their expression dynamics during liver development, i.e., When they start their liver expression and when they stop liver expression?

Response: Thank you for your valuable comments. We have added data on the expressions of *khdrbs1a* and *khdrbs1b* from 56 hpf (hours post fertilization) to 5 dpf, respectively. Apparently, *khdrbs1a* and *khdrbs1b* were expressed during early liver development but ceased expression at 4 dpf and 5 dpf (Fig. S4A in the revised Supplementary Information).

9. Fig. 2D, lower panel: Does anti-GFP indicate Tg(Tp1: GFP) signal (labeling intrahepatic BECs)? Why there is no GFP signal (= no BECs?) in the control embryo?

Response: We thank the reviewer for the insightful questions. 1) We have added the details of the transgenic fishes in Fig. 2B of the revised Figure 2. The transgenic line *Tg(TRE3G: dCas9NLS-T2A-GFP; U6: gRNA-khdrbs1; cryaa: venus; krt18: Tet-on 3G)* was used. The GFP signal represents the expression of dCas9, which is driven by Tet-on 3G/TRE3G system in *krt18*$^{+}$ BECs after Dox treatment. 2) There is no GFP signal in the control group due to no *Tet-on 3G* driven by *krt18* promoter after Dox treatment.

10. "The regenerating livers of dCas9iS subgroup co-expressed GFP and Dendra2 at R48h, demonstrating that this dCas9i system is BECs-specific for biliary-mediated liver regeneration (Fig. 2D, bottom panel)": This sentence is difficult to understand, why co-expression of GFP and Dendra2 in the regenerating livers could demonstrate the dCas9i system is BECs-specific?

Response: Thank you for your kind question. We have revised this point using the 2-dimensional pictures (Fig. 2C in the revised Figure 2). GFP expression was induced in *krt18*$^{+}$ BECs by Dox treatment, suggesting the expression of dead Cas9 protein. Co-expression of GFP and Dendra2 indicated that dead Cas9 is expressed and works in regenerating livers of the dCas9iS group.

11. The subtitle "Specific knockdown of khdrbs1 in BECs results in impaired biliary-mediated liver regeneration and activated p53 signaling": However, no experimental data concerning p53 signaling were mentioned in this section.

Response: We thank the reviewer for pointing this out. We have deleted the irrelevant content in this subtitle in the revised manuscript.

12. "khdrbs1 mutants exhibited a significant upregulation of tp53, mdm2, and ccng1 across broad anatomical regions including optic tectum, pharyngeal pouches, and gut, but not in the liver at 5 dpf (Fig. S5A)": It's interesting that the expression of tp53, mdm2, and ccng1 is activated in some specific embryonic regions but not liver in the khdrbs1 mutants, does it mean khdrbs1a and khdrbs1b are not expressed in liver at 5 dpf? Will tp53, mdm2, and ccng1 expression be induced in khdrbs1 mutant liver at 3.5 dpf, when khdrbs1a and khdrbs1b are present in WT liver at this stage (and might be required to repress p53 in liver at this stage)?

Response: Thank you for raising this interesting question. 1) We have included additional data on the expressions of *khdrbs1a* and *khdrbs1b* in wild-type larvae at 5 dpf. Apparently, *khdrbs1a* and *khdrbs1b* are not expressed in the liver at 5 dpf (Fig. S4A in the revised Supplementary Information). 2) p53 associated genes *tp53*, *mdm2* and *ccng1* were detected in *khdrbs1*$^{-/-}$ mutant at 3.5 dpf. WISH data shown that these genes were upregulated in brain and intestine of *khdrbs1*$^{-}$

© 2025. Published by The Company of Biologists under the terms of the Creative Commons Attribution License (https://creativecommons.org/licenses/by/4.0/).

$^{/-}$ mutant, but not significantly expressed in liver (Fig. S8A in the revised Supplementary Information). In controls, *khdrbs1* and *khdrbs1b* were expressed in liver at 3.5 dpf (Fig. S4D and S4E in the revised Supplementary Information), whereas p53 associated genes were not expressed. However, these genes were not expressed in the liver of controls at 5 dpf (Fig. S4A and S8A in the revised Supplementary Information). These data suggest that there may be a low correlation between Khdrbs1 and p53 during liver development.

13. Fig. S5C vs Fig. 4A: Why tp53 was not upregulated as shown by q-PCR (Fig. S5C) but increased in the liver as shown by WISH (Fig. 4A)?

Response: We thank the reviewer for raising this important question. Notably, q-PCR data showed that *Δ113tp53*, but not *tp53*-full length *(tp53-FL)*, was upregulated in *khdrbs1*$^{-/-}$ mutant (Fig. S8C in the revised Supplementary Information). *Δ113tp53* is a variant of *tp53* produced by an alternative internal promoter in the *tp53* gene and has been reported as a target gene of p53 in zebrafish (Chen et al., 2009; Guo et al., 2010). Our RNA probe of *tp53* used in WISH could not distinguish between these two products of *tp53* (Fig. 4A in the revised figure 4 and Fig. S8B in the revised Supplementary Information). However, the q-PCR method is more sensitive to detect these two *tp53* products using specific primers.

14. "Further investigations of WISH analyses indicated that tp53 and mdm2 were significantly upregulated in the regenerating liver of khdrbs1 mutants at R8h (Fig. S5B)": p53 signaling upregulation seems quite early in khdrbs1 mutants after liver injury. How early is it activated? Khdrbs1 seem to be responsible to repress p53 activation during the liver regeneration. Is there any correlation on the timing between khdrbs1 upregulation in WT embryos and p53 signaling activation in khdrbs1 mutants?

Response: Thank you for your helpful comments. 1) To answer the point-in-time of p53 activated, we used our specific knockdown method to check the expression of p53 associated genes, *mdm2* and *ccng1*, at R0h and R8h, respectively. WISH results indicated that *mdm2* and *ccng1* were apparently expressed in liver region at R8h rather than R0h after hepatocyte ablation (Fig. S9A and S9B in the revised Supplementary Information), indicating that p53 is activated after R0h, especially at R8h. We also evaluated *tp53* expression via FISH and p53 antibody staining at R8h, suggesting that p53 has high expression in BPPCs of the *khdrbs1*$^{-/-}$ mutant (Fig. 4B in the revised manuscript and Fig. S9C in the revised Supplementary Information). 2) Additionally, our data show that p53 signaling is active and functioning around R8h, which corresponds to the time at which khdrbs1 begins to be highly expressed. This suggests that p53 indeed responds to the loss of Khdrbs1 during liver regeneration.

15. Fig. 4B-4F: The authors should provide the result of tp53+/- as a control.

Response: We thank the reviewer for this valuable suggestion. We have provided the data of *tp53*$^{+/-}$ larvae (Fig. 4C-4G in the revised Figure 4) in the revised manuscript and figures.

16. "the proliferation rate of BPPCs was partially rescued by the heterozygous tp53 (tp53+/-) in khdrbs1 mutants (Fig. 4B)": However, this figure showed the ratio of EdU+ cells among Dendra2+ cells (representing hepatocytes), which cannot demonstrate proliferation of BPPCs.

Response: We thank the reviewer for this insightful comment. We have supplemented the channel of anti-Anxa4 and recalculated the percentage of EdU^{+} cells among Anxa4 and Dendra2 co-expressed cells, which represent BPPCs at R24h (Fig. 4C in the revised Figure 4 and manuscript).

17. Heterozygous tp53 (tp53+/-) partially rescued liver regeneration defects of the khdrbs1 mutants (Fig. 4B-4F), could p53 homozygous mutant give better rescue effects?

© 2025. Published by The Company of Biologists under the terms of the Creative Commons Attribution License (https://creativecommons.org/licenses/by/4.0/).

Response: We thank the reviewer for this constructive comment. We have further evaluated the redifferentiation and liver size of *khdrbs1*$^{-/-}$;*tp53*$^{-/-}$ mutant. The *tp53* homozygousmutationalsopartiallyrescuedthedefectivephenotypes of*khdrbs1*$^{-/-}$ mutant, but the effect was comparable to that observed with the heterozygous *tp53* mutation (Fig. S10A-S10D in the revisedSupplementary Information).

Minor comments:

1. Fig. 1A, 1D, 2B, 3A, 3D, 4B, S2A, S3A, S4A: What does "BT" mean?

Response: Thank you for your kind reminder. “BT” stands for before treatment with Mtz. We have now explicitly indicated this in the revised manuscript.

2. Fig. 1B: The color of the scale bar (from 0.0 to 9.0) starts with orange (0.0) and ends with red (9.0), does it represent relative level of gene expression and 0.0 means no expression? If yes, why there are grey-colored cells in the UMAP?

Response: We thank the reviewer for this insightful comment. The color scale indeed represents the relative level of gene expression. Grey-colored cells indicate that the gene expression is close to 0.0, rather than completely absent, suggesting a lower expression level compared to other cells.

3. Fig. 1B: "Lgo2 Exp" seems should be "Log2 Exp"?

Response: Thank you for pointing out this error. We have corrected it to "Log2 Exp" in the revised manuscript (Fig. 1B in the revised Figure 1).

4. Fig S2A: (1) Indicate the transgenic fish used? (2) Right panel: "Lver area at R48h" should be "Liver area at R48h"?

Response: We thank the reviewer for pointing out these issues. (1) We have indicated the transgenic fish and time points (Fig S5A in the revised Supplementary Information). (2) We have corrected the spelling error from “Lver” to “Liver” (Fig. S5A in the revised Supplementary Information).

5. "The expressions of tp53, mdm2 and ccng1 were also highly increased in the regenerating livers of khdrbs1 mutants at R24h (Fig. 5A)": Fig. 5A should be Fig. 4A?

Response: We thank the reviewer for pointing out this error and apologize for the oversight. “Fig. 5A” have been corrected to “Fig. 4A” in the revised manuscript.

6. "the proportion of Alcam+ cells among Dendra2+ cells was diminished by tp53 mutation in khdrbs1 mutants (Fig. 5C and D), indicating that the redifferentiation of BPPCs was rescued. Moreover, tp53 mutation also partially rescued the regenerating liver size of khdrbs1 mutants at R48h (Fig. 5E), as well as the expression of hepatocyte markers bhmt and hnf4a (Fig. 5F)": Fig. 5C through 5F should be Fig. 4 C through 4F?

Response: We sincerely thank the reviewer for highlighting this issue and apologizeonceagainforthisoversight.“Fig.5”havebeencorrectedto“Fig.4” intherevisedmanuscript.

7. English grammar needs improvements, e.g., "khdrbs1a was decreased expression in regenerating livers of dCas9i group".

Response: Thank you for your valuable feedback. We have corrected this misrepresentation in the

© 2025. Published by The Company of Biologists under the terms of the Creative Commons Attribution License (https://creativecommons.org/licenses/by/4.0/).

revised manuscript and have improved the overall English grammar throughout the text.

8. The citation of some reference is incomplete, e.g., the one of Fu, K. et al.

Response: Thank you for your careful reviewing. We have thoroughly checked all references and revised them to meet the citation criteria of *Development*.

References:

Cai,P.,Mao,X.,Zhao,J.,Nie,L.,Jiang,Y.,Yang,Q.,Ni,R.,He,J.,Luo,L. (2021).FarnesoidXReceptorIs RequiredfortheRedifferentiationof BipotentialProgenitorCellsDuringBiliary-Mediated ZebrafishLiver Regeneration.*Hepatology***74**,3345-3361.

Chen,J.,Ruan,H.,Ng,S.M.,Gao,C.,Soo,H.M.,Wu,W.,Zhang,Z.,Wen, Z.,Lane,D.P.,Peng,J.(2005). Lossoffunctionofdefselectivelyup- regulatesDelta113p53expressiontoarrestexpansion growthof digestiveorgansinzebrafish.*GenesDev***19**,2900-2911.

Chen,J.,Ng,S.M.,Chang,C.,Zhang,Z.,Bourdon,J.C.,Lane,D.P.,Peng, J.(2009).p53isoform delta113p53isap53targetgenethat antagonizesp53apoptoticactivityviaBclxLactivationin zebrafish. *GenesDev***23**,278-290.

Choi,T.Y.,Ninov,N.,Stainier,D.Y.,Shin,D.(2014).Extensiveconversionof hepaticbiliaryepithelial cellstohepatocytesafterneartotallossof hepatocytesinzebrafish.*Gastroenterology***146**,776-788.

Guo,L.,Chua,J.,Vijayakumar,D.,Lee,K.C.,Lim,K.,Eng,H.,Ghadessy, F.,Coomber,D.,Lane,D.P. (2010).Detectionofthe113p53protein isoform:ap53-inducedproteinthatfeedsbackonthe p53pathwayto modulatethep53responseinzebrafish.*CellCycle***9**,1998-2007.

He,J.,Lu,H.,Zou,Q.,Luo,L.(2014).Regenerationofliverafterextreme zebrafish.*Gastroenterology***146**,789-800.

He,J.,Zhou,Y.,Qian,C.,Wang,D.,Yang,Z.,Huang,Z.,Sun,J.,Ni,R., Yang,Q.,Chen,J.,Luo,L. (2022).DNAmethylationmaintenanceat thep53locusinitiatesbiliary-mediatedliver regeneration.*NPJRegen Med***7**,21.

Ninov,N.,Borius,M.,Stainier,D.Y.R.(2012).DifferentlevelsofNotch signalingregulatequiescence, renewalanddifferentiationinpancreatic endocrineprogenitors.*Development***139**,1557-1567.

Okamoto,K.,Beach,D.(1994).Cyclin-GIsaTranscriptionalTargetofthe P53Tumor-SuppressorProtein. *EmboJournal***13**,4816-4822.

Song,J.,Ma,J.,Liu,X.,Huang,Z.,Li,L.,Li,L.,Luo,L.,Ni,R.,He,J. (2023).TheMRNcomplexmaintains thebiliary-derivedhepatocytesin liverregenerationthroughATR-Chk1pathway.*NPJRegen Med***8**,20.

Second decision letter

MS ID#: dev.204266R1

MS TITLE: Khdrbs1 drives redifferentiation of bipotential progenitor cell by inhibiting p53 in zebrafish biliary-mediated liver regeneration

AUTHORS: Kai Gang, Qi Chen, Junhui Sun, Pengcheng Cai, Rui Ni and Jianlong Ma

I have now received all the referees reports on the above manuscript, and have reached a decision. The referees' comments are appended below, or you can access them online: please go to .

We would like to publish a revised manuscript in Development, provided that the referees' comments can be satisfactorily addressed. Please attend to all of the reviewers' comments in your

© 2025. Published by The Company of Biologists under the terms of the Creative Commons Attribution License (https://creativecommons.org/licenses/by/4.0/).

revised manuscript and detail them in your point-by-point response. Reviewers #1 and #2 were positive and only minor revisions were accepted. Reviewer #3 had several concerns. In considering this, I have decided to move forward and ask you to address both #1 and #3 comments in writing, by incorporating appropriate edits and clarifications into the Results and Discussion sections of your manuscript. If you do not agree with any of their criticisms or suggestions explain clearly why this is so in your response letter.

Comments from the Reviewers:

Reviewer 1: The revised manuscript is much improved and addresses most of my comments. I have the following minor comments, which can be easily addressed by the authors.

1. Given that tp53 homo still partially rescued the liver regeneration defects observed in khdrbs1 mutants, it is better to tone down the conclusion of the manuscript by adding "in part". For example, we elucidate that Khdrbs1 promotes BPPC redifferentiation in part by inhibiting p53 activation during biliary-mediated liver regeneration in zebrafish (lines 30-32).

2. The rationale for investigating p53 signaling is still not clear (lines 233-234). Please rewrite the sentence.

3. Could you elaborate on how khdrbs1 deficiency activates p53 signaling in the regenerating livers? Given the strong expression/induction of both tp53 mRNA and protein in BPPCs of khdrbs1 mutants, the statement that "Khdrbs1 may indirectly influence p53 stability (line 275)" is too weak.

4. Provide an allele name of the Tg(krt18:Tet-on3G;cryaa:CFP;TRE3G:dCas9NLS-T2A-GFP;U6:gRNA-khdrbs1a;cryaa:venus) line.

Reviewer 2: This is a thoroughly revised manuscript. I would like to congratulate the authors to have invested a lot of time, resources, new data, and more refined introduction plus discussion sections to make this a compelling piece of work. I have no further concerns.

Reviewer 3: 1. The revised manuscript provided more details on the previous experiments and results, however, the most critical issue still remains, i.e., the mechanism on how Khdrbs1 repress p53 signaling is still missing, and no new data are provided toward this aspect. I think current version is still too simple/superficial to publish as a decent Development paper.
2. The abnormal expression of p53 in the khdrbs1 mutants might not be the primary effect of Khdrbs1 loss-of-function, since rescue of tp53 mutation on liver regeneration defects of khdrbs1 mutants is only evident at later stage (R24h) but not early stage (R8h), and only showed partial rescue rather than fully rescue. The p53 effect is more likely a further down-stream secondary effect. Demonstrating a direct interaction between Khdrbs1 and p53 or revealing an immediate down-stream target of Khdrbs1 is necessary to solve this issue.
3. There are not enough data to support the claim that the function of Khdrbs1 is to promote redifferentiation of BPPCs during biliary-mediated liver regeneration, since one cannot exclude other possibilities, e.g., Khdrbs1 may be able to promote hepatocyte lineage cell proliferation rather than to promote redifferentiation, or the liver regeneration defects may be due to developmental defects of khdrbs1 mutants on normal hepatocyte differentiation (which leads to smaller liver in BT samples, Fig. 3D), etc.
4. INTRODUCTION is fairly improved, however, some more recent or relevant publications are not cited and mentioned (e.g., PMID: 38660302, 26438629, 27365047, 27690217, 38351330, 38454028, 29354154, etc), especially those concerning interactions/correlations between Khdrbs1/Sam68 and p53. And the authors should carefully compare and discuss their work with the most relevant publication regarding the relations between Khdrbs1 and p53.

© 2025. Published by The Company of Biologists under the terms of the Creative Commons Attribution License (https://creativecommons.org/licenses/by/4.0/).

5. "BECs proliferate and facilitate recovery from severe liver injury by dedifferentiating into bipotential progenitor cells (BPPCs)" (lines 70-71), "BPPCs expressed hepatobalsts markers such as sox9b (SRY-box transcription factor 9b), foxa3 (forkhead box A3), and hhex (hematopoietically expressed homeobox)" (lines 73-75): These information indicate that BEC and BPPC are two different cell types. In the R0h liver, only BPPC but no BEC cell population was identified by scRNA-seq (Fig. 1B), however, the authors claim "khdrbs1a and khdrbs1b are expressed in BECs at R0h" in Fig. S2. Where exactly khdrbs1a and khdrbs1b are expressing?
6. Fig. 2D, 3E, 4A, 4G, S6D, and S6E: How is the expression of these markers in un-injured (no Mtz treatment) embryos?
7. Fig. S3D: (1) What is the percentage of khdrbs1a- or khdrbs1b-expressing cells in GFP+&Dendra2- cells? (2) What is the percentage of Dendra2+ cells in GFP+ cells? (3) What is the percentage of GFP+ cells in Dendra2+ cells?
8. Fig. S4B and S4C: It will be more informative if the authors could depict the structure of the expected protein products of WT and khdrbs1 mutants.
9. "khdrbs1a-/- or khdrbs1b-/- mutants showed normal liver regeneration after hepatocyte ablation (Fig. S5A), suggesting the genetic compensation" (lines 144-146): This speculation is not accurate. It might also be due to redundancy.
10. Fig. S8: How are the expression of these genes in your scRNA-seq data?
11. The single-cell RNA sequencing data of lfabp, Dendra2, her9 and anxa4 are informative and helpful, better include in the manuscript. Would also be helpful to include the data of krt18 and p53 expression.
12. "total RNA was extracted from 30-50 stripped livers" (lines 392-393): The authors should describe how the livers were stripped.
13. "we introduced tp53M214K into the khdrbs1-/- mutant" (lines 253-254): A brief introduction of tp53M214K should be provided.
14. "To evaluate the expressions of hepatoblast markers and p53-associated genes, total RNA was extracted from 30 whole embryos" (lines 394-396): Why use whole embryos instead of livers in these experiments?
15. "However, the expressions of tp53, mdm2, and ccng1 were significantly increased in the regenerating livers of khdrbs1-/- mutants (Fig. S8B)" (lines 244-246): (1) What does "However" mean here? (2) Better indicate the stage of the embryos.
16. The information about p53 in situ probe and its relationship with Î”113tp53 and tp53-FL is important and should be included in the manuscript.
17. The academic name of zebrafish need to be given at its first appearance in the main text.
18. "indicating the defective liver regeneration after knockdown of khdrbs1 (Fig. 2D)" (lines 166-167): "khdrbs1" should be "khdrbs1a"?
19. Tg(lfabp: CFP-NTR) fish line is mentioned in MATERIALS AND METHODS, however, no experiments were performed on this line.

Second revision

Author response to reviewers' comments

Point by Point Response to the Reviewers

Response to Reviewer #1

Reviewer 1: The revised manuscript is much improved and addresses most of my comments. I have the following minor comments, which can be easily addressed by the authors.
Response: We sincerely thank the reviewer for the positive feedback and helpful suggestions, which have greatly improved our manuscript. We have carefully addressed all your comments and revised the manuscript accordingly.

1. Given that tp53 homo still partially rescued the liver regeneration defects observed in khdrbs1 mutants, it is better to tone down the conclusion of the manuscript by adding "in part". For

© 2025. Published by The Company of Biologists under the terms of the Creative Commons Attribution License (https://creativecommons.org/licenses/by/4.0/).

example, we elucidate that Khdrbs1 promotes BPPC redifferentiation in part by inhibiting p53 activation during biliary-mediated liver regeneration in zebrafish (lines 30-32).
Response: We sincerely thank the reviewer for this constructive suggestion. We agree with your point and have made the suggested adjustment in the revised manuscript to tone down the conclusion accordingly (lines 28).

2. The rationale for investigating p53 signaling is still not clear (lines 233-234). Please rewrite the sentence.
Response: We sincerely thank the reviewer for pointing out this issue. We have revised the sentence and clarified the rationale for investigating p53 signaling in the revised manuscript (lines 234-239).

3. Could you elaborate on how khdrbs1 deficiency activates p53 signaling in the regenerating livers? Given the strong expression/induction of both tp53 mRNA and protein in BPPCs of khdrbs1 mutants, the statement that "Khdrbs1 may indirectly influence p53 stability (line 275)" is too weak.
Response: We thank the reviewer for these valuable comments. As previously reported, Mdm4 is a known negative regulator of p53 activity (Almeida et al., 2022; Wu et al.,2021), and recent study suggest that Khdrbs1 (Sam68) may influence the alternative splicing of Mdm4 (Cai et al., 2021). In this study, we observed that the loss of Khdrbs1 affects the alternative splicing of Mdm4, resulting in decreased expression of Mdm4-FL (Fig R1. A-D). These findings suggest that Khdrbs1 might regulate p53 signaling by modulating Mdm4 splicing. We acknowledge that confirming this hypothesis requires additional experiments, such as generating relevant mutant and overexpression lines, which cannot be completed within the timeframe of this study. Due to the graduation timeline of the first author, we plan to submit the current work as a Research Report to *Development* and address this critical question in detail in future studies. Additionally, the strict word limit of the Research Report format prevented us from including these findings in the manuscript, but we would be happy to share these data (Fig R1. A-D) with you personally.
Furthermore, we have revised the statement that "Khdrbs1 may inhibit the p53 activity rather than function as a coactivator of p53 (Li and Richard, 2016)" (lines 288-289).

NOTE: We have removed unpublished data that had been provided for the referees in confidence.

Fig R1. The expression of *mdm4-FL* is downregulated after loss of *khdrbs1* in regenerating liver at R0h. (A) PCR and the quantificational expression analysis of the alterative splicing levels of *mdm4* in the regenerating liver of control and dCas9i groups at R0h after Dox treatment, respectively. *hhex* is a marker of the regenerating liver at R0h. (B) The quantificational expression levels of *hhex* and the alterative splicing of *mdm4* (*mdm4-S*) in the regenerating liver of control and dCas9i groups at R0h, respectively. (C) The ratio of full-length transcript of *mdm4* (*mdm4-FL*) expression in the regenerating livers of control and dCas9i groups at R0h. (D) Single-optical section images via FISH and antibody staining showing the *mdm4* expression (green) in Anxa4$^+$ BPPCs (red) of the control and *khdrbs1*$^{-/-}$ mutant at R0h. Normalized with the expression levels of *eef1a1l* in PCR and the quantification. ns: no significantly different. Asterisks indicate statistical significance: *$p < 0.05$. n: the technical replicates. Data are represented as mean±SEM. Scale bar: 50 μm.

4. Provide an allele name of the Tg(krt18:Tet-on3G;cryaa:CFP;TRE3G:dCas9NLS-T2A-GFP;U6:gRNA-khdrbs1a;cryaa:venus) line.
Response: We thank the reviewer for the careful comment. The allele name for this transgenic line is *Tg(krt18:Tet-on3G;TRE3G:dCas9NLS;U6:gRNA-khdrbs1a)*. This transgenic line was generated by crossing the *Tg(krt18:Tet-on 3G;cryaa:CFP)* line with the *Tg(TRE3G:dCas9NLS-T2A-GFP;U6:gRNA-khdrbs1a;cryaa:venus)* line.

Response to Reviewer #2

Reviewer 2: This is a thoroughly revised manuscript. I would like to congratulate the authors to have invested a lot of time, resources, new data, and more refined introduction plus discussion sections to make this a compelling piece of work. I have no further concerns.

© 2025. Published by The Company of Biologists under the terms of the Creative Commons Attribution License (https://creativecommons.org/licenses/by/4.0/).

Response: We sincerely thank the reviewer for the time to evaluate our manuscript and for the positive comments on our revision.

Response to Reviewer #3

Reviewer 3: 1. The revised manuscript provided more details on the previous experiments and results, however, the most critical issue still remains, i.e., the mechanism on how Khdrbs1 repress p53 signaling is still missing, and no new data are provided toward this aspect. I think current version is still too simple/superficial to publish as a decent Development paper.
Response: We sincerely thank the reviewer for this valuable and insightful comment. As previously reported, Mdm4 is a well-established negative regulator of p53 activity (Almeida et al., 2022; Wu et al.,2021), and recent study suggest that Khdrbs1 (Sam68) may influence the alternative splicing of Mdm4 (Cai et al., 2021). Here, we observed that the loss of Khdrbs1 affects the alternative splicing of Mdm4, resulting in decreased expression of Mdm4-FL (Fig R1. A-D). These findings suggest that Khdrbs1 might regulate p53 signaling by modulating Mdm4 splicing. However, validating this hypothesis requires additional experiments, such as the generation of relevant mutant and overexpression lines, which cannot be completed within the timeframe of this study. Due to the graduation timeline of the first author, we plan to submit the current work as a Research Report to *Development* and address this critical question in detail in future studies. Additionally, the strict word limit of the Research Report format prevented us from including these findings in the manuscript, but we would be happy to share these data (Fig R1. A-D) with you personally upon request. We greatly appreciate the reviewer's insightful guidance, which provides invaluable direction for our future work.

NOTE: We have removed unpublished data that had been provided for the referees in confidence.

Fig R1. The expression of *mdm4-FL* is downregulated after loss of *khdrbs1* in regenerating liver at R0h. (A) PCR and the quantificational expression analysis of the alterative splicing levels of *mdm4* in the regenerating liver of control and dCas9i groups at R0h after Dox treatment, respectively. *hhex* is a marker of the regenerating liver at R0h. (B) The quantificational expression levels of *hhex* and the alterative splicing of *mdm4* (*mdm4-S*) in the regenerating liver of control and dCas9i groups at R0h, respectively. (C) The ratio of full-length transcript of *mdm4* (*mdm4-FL*) expression in the regenerating livers of control and dCas9i groups at R0h. (D) Single-optical section images via FISH and antibody staining showing the *mdm4* expression (green) in Anxa4$^+$ BPPCs (red) of the control and *khdrbs1*$^{-/-}$ mutant at R0h. Normalized with the expression levels of *eef1a1l* in PCR and the quantification. ns: no significantly different. Asterisks indicate statistical significance: **p* < 0.05. n: the technical replicates. Data are represented as mean±SEM. Scale bar: 50 µm.

2. The abnormal expression of p53 in the khdrbs1 mutants might not be the primary effect of Khdrbs1 loss-of-function, since rescue of tp53 mutation on liver regeneration defects of khdrbs1 mutants is only evident at later stage (R24h) but not early stage (R8h), and only showed partial rescue rather than fully rescue. The p53 effect is more likely a further down-stream secondary effect. Demonstrating a direct interaction between Khdrbs1 and p53 or revealing an immediate down-stream target of Khdrbs1 is necessary to solve this issue.
Response: We appreciate the reviewer's helpful and constructive comment. We agree that the p53 signaling pathway may be indirectly activated in the absence of Khdrbs1. Regarding the immediate downstream targets of Khdrbs1, we have conducted some preliminary investigations (Fig. R1 A-D) but have not yet reached definitive conclusions (see our response to comment #1). Further experiments will be required to fully address this issue. We sincerely thank the reviewer for their insightful guidance, which will greatly benefit our future research.

3. There are not enough data to support the claim that the function of Khdrbs1 is to promote redifferentiation of BPPCs during biliary-mediated liver regeneration, since one cannot exclude other possibilities, e.g., Khdrbs1 may be able to promote hepatocyte lineage cell proliferation rather than to promote redifferentiation, or the liver regeneration defects may be due to

© 2025. Published by The Company of Biologists under the terms of the Creative Commons Attribution License (https://creativecommons.org/licenses/by/4.0/).

developmental defects of khdrbs1 mutants on normal hepatocyte differentiation (which leads to smaller liver in BT samples, Fig. 3D), etc.
Response: We thank the reviewer for raising this insightful question and giving us the opportunity to clarify these points. In our zebrafish severe liver injury model, when hepatocytes are extremely damaged, BECs first dedifferentiate into BPPCs and subsequently redifferentiate into nascent hepatocytes and BECs to facilitate liver regeneration (He et al., 2014). Additionally, our previous study has shown that the undifferentiated BPPCs can be marked by Dendra2$^+$/Alcam$^+$ and Hnf4α$^+$/Alcam$^+$, and that the accumulation of BPPCs is a hallmark of redifferentiation defects (Cai et al., 2021; Ma et al., 2023; Zhang et al., 2024). In this study, we observed that compared to controls, *khdrbs1* mutants exhibited a significant accumulation of Dendra2$^+$/Alcam$^+$ and Hnf4α$^+$/Alcam$^+$ cells (Fig. 3F,G), suggesting the maintenance of these regenerating cells at the bipotential state rather than redifferentiation into mature hepatocytes. These findings indicate defective redifferentiation in *khdrbs1* mutants, resulting in the accumulation of BPPCs.
1) While we cannot entirely exclude the possibility that Khdrbs1 promotes hepatocyte lineage cell proliferation rather than redifferentiation, this hypothesis does not explain the observed accumulation of BPPCs, which is a well-established hallmark of redifferentiation defects.
2) We agree with the reviewer that liver developmental defects in *khdrbs1* mutants could potentially contribute to regeneration impairment. To specifically address this, we utilized a BEC-specific knockdown system to deplete *khdrbs1* in biliary epithelial cells. Our results demonstrate that the loss of *khdrbs1* in BECs alone is sufficient to cause defects in BEC-mediated liver regeneration (Fig. 2). This finding helps exclude developmental defects as the primary cause of the observed regeneration phenotype.

4. INTRODUCTION is fairly improved, however, some more recent or relevant publications are not cited and mentioned (e.g., PMID: 38660302, 26438629, 27365047, 27690217, 38351330, 38454028, 29354154, etc), especially those concerning interactions/correlations between Khdrbs1/Sam68 and p53. And the authors should carefully compare and discuss their work with the most relevant publication regarding the relations between Khdrbs1 and p53.
Response: We thank the reviewer for pointing out these important references. As our manuscript was submitted as a Research Report to *Development*, which has a strict word limit, we regret that it was not feasible to include all of the cited publications. We have now included citations to the following publications: PMID: 38351330, 27365047, 38660302, and 26438629 (lines 81-86). Additionally, we have compared and discussed our findings in relation to these studies (lines 288-289).

5. "BECs proliferate and facilitate recovery from severe liver injury by dedifferentiating into bipotential progenitor cells (BPPCs)" (lines 70-71), "BPPCs expressed hepatobalsts markers such as sox9b (SRY-box transcription factor 9b), foxa3 (forkhead box A3), and hhex (hematopoietically expressed homeobox)" (lines 73-75): These information indicate that BEC and BPPC are two different cell types. In the R0h liver, only BPPC but no BEC cell population was identified by scRNA-seq (Fig. 1B), however, the authors claim "khdrbs1a and khdrbs1b are expressed in BECs at R0h" in Fig. S2. Where exactly khdrbs1a and khdrbs1b are expressing?
Response: We sincerely thank the reviewer for carefully pointing out this issue. While BPPCs and BECs are indeed two distinct cell types, BPPCs derived from BECs can still express certain markers characteristic of BECs. To address this and improve clarity, we have revised the statement "*khdrbs1a* and *khdrbs1b* are expressed in BECs at R0h" to "*khdrbs1a* and *khdrbs1b* are expressed in BPPCs at R0h" in Fig. S3 of Supplementary information.

6. Fig. 2D, 3E, 4A, 4G, S6D, and S6E: How is the expression of these markers in un-injured (no Mtz treatment) embryos?
Response: We thank the reviewer for this constructive comment. We have now included the expression profiles of these markers in uninjured livers of the control and *khdrbs1*$^{-/-}$ mutant embryos (Fig. S8A,B).

© 2025. Published by The Company of Biologists under the terms of the Creative Commons Attribution License (https://creativecommons.org/licenses/by/4.0/).

7. Fig. S3D: (1) What is the percentage of khdrbs1a- or khdrbs1b-expressing cells in GFP+&Dendra2- cells? (2) What is the percentage of Dendra2+ cells in GFP+ cells? (3) What is the percentage of GFP+ cells in Dendra2+ cells?
Response: We thank the reviewer for these detailed questions. We believe that the data presented in Fig. S3D are sufficient to support our finding that *khdrbs1a* is mainly expressed in the BEC-derived cells (GFP^+&$Dendra2^+$ cells), as raised in the reviewer's comment during the first round of review. These data already include the percentage of GFP^+ or $Dendra2^+$ cells. Notably, GFP^+&$Dendra2^-$ cells were rarely observed in these experiments. We also observed that *khdrbs1b* was mainly expressed in the GFP^+&$Dendra2^+$ cells but not in other cells. To address the potential confusion caused by the insufficient clarity of the previous Y-axis labels, we have revised the Y-axis labels in Fig. S4D to make them clearer (Fig. S4D).

8. Fig. S4B and S4C: It will be more informative if the authors could depict the structure of the expected protein products of WT and khdrbs1 mutants.
Response: We thank the reviewer for this constructive suggestion. We have now included diagrams of the predicted protein structures of both WT and *khdrbs1* mutant (Fig. S5B and S5C).

9. "khdrbs1a-/- or khdrbs1b-/- mutants showed normal liver regeneration after hepatocyte ablation (Fig. S5A), suggesting the genetic compensation" (lines 144-146): This speculation is not accurate. It might also be due to redundancy.
Response: We agree with the reviewer that "redundancy" is a reasonable explanation. The text has been revised as follows: "*khdrbs1a*$^{-/-}$ or *khdrbs1b*$^{-/-}$ mutants showed normal liver regeneration after hepatocyte ablation (Fig. S6A,B), suggesting potential genetic compensation or functional redundancy between the two paralogues" (lines 144-145).
10. Fig. S8: How are the expression of these genes in your scRNA-seq data?
Response: We thank the reviewer for this valuable comment. We have incorporated the single-cell RNA sequencing data of these genes in the revised Supplementary Information (Fig. S2A-C).

11. The single-cell RNA sequencing data of lfabp, Dendra2, her9 and anxa4 are informative and helpful, better include in the manuscript. Would also be helpful to include the data of krt18 and p53 expression.
Response: We thank the reviewer for this insightful suggestion. we have included the single-cell RNA sequencing data of these genes in the revised Supplementary Information Fig. S2B and Fig. S10A.

12. "total RNA was extracted from 30-50 stripped livers" (lines 392-393): The authors should describe how the livers were stripped.
Response: We thank the reviewer for pointing this out. We have added a description of the liver stripping method in the revised manuscript. "total RNA was extracted from 30-50 livers stripped with fine forceps in PBS" (lines 408-411).

13. "we introduced tp53M214K into the khdrbs1-/- mutant" (lines 253-254): A brief introduction of tp53M214K should be provided.
Response: We thank the reviewer for raising this important issue. We have added a brief introduction of *tp53*^{M214K} in the revised manuscript (lines 266-267).

14. "To evaluate the expressions of hepatoblast markers and p53-associated genes, total RNA was extracted from 30 whole embryos" (lines 394-396): Why use whole embryos instead of livers in these experiments?
Response: We thank the reviewer for carefully reading our manuscript and raising this important point. Due to the difficulty in distinguishing mutants at R8h, and the small size of the regenerating liver, particularly at R24h, it was challenging to collect sufficient liver tissue. Therefore, we initially used whole embryos to extract total RNA. However, we agree that using liver tissue is more appropriate than using whole embryos and have now repeated the qPCR experiments using stripped liver tissues (lines 412-414; Fig. S7C; Fig. S10E).

© 2025. Published by The Company of Biologists under the terms of the Creative Commons Attribution License (https://creativecommons.org/licenses/by/4.0/).

15. "However, the expressions of *tp53*, *mdm2*, and *ccng1* were significantly increased in the regenerating livers of khdrbs1-/- mutants (Fig. S8B)" (lines 244-246): (1) What does "However" mean here? (2) Better indicate the stage of the embryos.
Response: We appreciate the reviewer for pointing out these issues. 1) The use of "However" was indeed incorrect in this context, as there is no contrast intended, and we have replaced "However" with "Moreover". 2) We agree that specifying the stage of the embryos improves clarity. We have added "R24h" to indicate the stage in this sentence. The revised sentence now reads: "Moreover, the expressions of *tp53*, *mdm2*, and *ccng1* were significantly increased in the regenerating livers of *khdrbs1*$^{-/-}$ mutants at R24h (Fig. S10D) " (lines 250-251).

16. The information about p53 in situ probe and its relationship with Δ113tp53 and tp53-FL is important and should be included in the manuscript.
Response: We thank the reviewer for this thoughtful suggestion. We have included the information about the p53 in situ probe and its relationship with *Δ113tp53* and *tp53-FL* in the revised manuscript (lines 252-256).

17. The academic name of zebrafish need to be given at its first appearance in the main text.
Response: We thank the reviewer for pointing this out. We have added the academic name of zebrafish, *Danio rerio*, at its first appearance in the INTRODUCTION section (line 59).

18. "indicating the defective liver regeneration after knockdown of khdrbs1 (Fig. 2D)" (lines 166-167): "khdrbs1" should be "khdrbs1a"?
Response: We thank the reviewer for thoroughly reviewing our manuscript and apologize for this mistake. We have corrected "*khdrbs1*" to "*khdrbs1a*" in the revised manuscript (line 166).

19. Tg(lfabp: CFP-NTR) fish line is mentioned in MATERIALS AND METHODS, however, no experiments were performed on this line.
Response: We appreciate the reviewer's careful observation. In our initial manuscript, the *Tg(lfabp:CFP-NTR)* fish line was used to provide additional characterization of the *khdrbs1* mutant phenotype. However, as our manuscript was submitted as a Research Report to *Development*, which has a strict word limit, we removed this part of the data to meet the submission requirements. Unfortunately, we neglected to remove this fish line in the MATERIALS AND METHODS section. We have now corrected this oversight by deleting the mention of this fish line.

Reference

Almeida G.M., Castilho A.C., Adamoski D., Braun-Prado K. (2022) MDM4: What do we know about the association between its polymorphisms and cancer? Med Oncol. **40**, 61.

Cai P, Mao X, Zhao J, Nie L, Jiang Y, Yang Q, Ni R, He J, Luo L. (2021) Farnesoid X Receptor Is Required for the Redifferentiation of Bipotential Progenitor Cells During Biliary-Mediated Zebrafish Liver Regeneration. Hepatology. 74, 3345-3361.

Cai, T., Cinkornpumin, J.K., Yu, Z., Villarreal, O.D., Pastor, W.A., Richard, S. (2021). Deletion of RBMX RGG/RG motif in Shashi-XLID syndrome leads to aberrant p53 activation and neuronal differentiation defects. *Cell reports* **36**, 109337.

Li, N., Richard, S. (2016). Sam68 functions as a transcriptional coactivator of the p53 tumor suppressor. *Nucleic acids research* **44**, 8726-8741.

He J, Lu H, Zou Q, Luo L. (2014) Regeneration of liver after extreme hepatocyte loss occurs mainly via biliary transdifferentiation in zebrafish. Gastroenterology. 146, 789-800.

Ma J, Yang Z, Huang Z, Li L, Huang J, Chen J, Ni R, Luo L, He J. (2023) Rngtt governs biliary-derived liver regeneration initiation by transcriptional regulation of mTORC1 and Dnmt1 in zebrafish. Hepatology. 78, 167-178.

Wu J., Lu G., Wang X. (2021) MDM4 alternative splicing and implication in MDM4 targeted cancer therapies. Am J Cancer Res. **11**, 5864-5880.

Zhang X, Liu H, Cai P, Huang Z, Ma J, Luo L. (2024) Mdka produced by the activated HSCs drives bipotential progenitor cell redifferentiation during zebrafish biliary-mediated liver regeneration. Hepatology. doi: 10.1097/HEP.0000000000001031. Epub ahead of print..

© 2025. Published by The Company of Biologists under the terms of the Creative Commons Attribution License (https://creativecommons.org/licenses/by/4.0/).

Third decision letter

MS ID#: dev.204266R2

MS TITLE: Khdrbs1 drives redifferentiation of bipotential progenitor cell by inhibiting p53 in zebrafish biliary-mediated liver regeneration

AUTHORS: Kai Gang, Qi Chen, Junhui Sun, Tingwei Zhang, Pengcheng Cai, Rui Ni and Jianlong Ma

I am happy to tell you that your manuscript has been accepted for publication in Development, pending our standard publication integrity checks. It was accepted on 26 Jan 2025. Where referee reports on this version are available, they are appended below.

© 2025. Published by The Company of Biologists under the terms of the Creative Commons Attribution License (https://creativecommons.org/licenses/by/4.0/).
